# Genome-Informed Characterization of *Lactiplantibacillus plantarum* ND88 with Inhibitory Activity against Representative Oral Pathogens

**DOI:** 10.1007/s12602-026-10992-1

**Published:** 2026-03-25

**Authors:** Hye Kim, Hyun Ha Lee, Sehyeon Song, Md Ariful Haque, Md Abdur Razzak, Min Ji Jang, Krithika Maki, Seockmo Ku

**Affiliations:** 1 Department of Food Science and Technology, Texas A&M University, College Station, TX USA; 2 Research Center, BIFIDO Co., Ltd, Hongcheon, 25117 South Korea

**Keywords:** Probiotics, Genome-informed analysis, Safety evaluation, GRAS, Adhesion and acid tolerance, Antibiotic resistance assessment, Oral pathogen inhibition

## Abstract

*Lactiplantibacillus plantarum* ND88, a human-derived strain with putative probiotic-associated traits, was taxonomically confirmed by whole-genome sequencing and comparative genomic analysis. Average nucleotide identity (ANI) analysis showed that ND88 clustered within the *L. plantarum* species group, sharing > 98% identity with representative reference genomes. Comparative pangenome analysis of ND88 and 20 *L. plantarum* genomes revealed multiple ND88-specific and enriched orthogroups, indicating strain-level genomic diversification. Genome-informed Pfam domain annotation predicted several adhesion-associated features, including repeated MucBP domains and predicted surface-associated proteins carrying LPxTG motifs and secretion signals. Consistent with this, *L. plantarum* ND88 exhibited approximately 2.2-fold greater adhesion to intestinal epithelial cells compared with *Lactocaseibacillus rhamnosus* GG. The strain exhibited strong acid tolerance, maintaining approximately 98% survival at pH 3. Genome mining using BAGEL4 identified a plantaricin bacteriocin biosynthetic locus, providing a genetic basis for antimicrobial activity. Correspondingly, ND88 inhibited representative oral pathogens, reducing adhesion by 29.8–57.4% and growth by 47.7–97.1% in co-culture assays. Genome-based safety assessment indicated the absence of virulence-associated genes and acquired antimicrobial resistance determinants. Although the minimum inhibitory concentrations for gentamicin and kanamycin were above EFSA microbiological cut-off values, no genomic evidence of acquired resistance was detected. ND88 also showed no hemolytic activity, the absence of bile salt hydrolase activity, and low D-lactate production (0.0068 g/L), comparable to GRAS-certified *L. plantarum* strains. These results demonstrate that ND88 is a genetically safe strain with experimentally supported probiotic-associated traits.

**Supplementary Information:**

The online version contains supplementary material available at 10.1007/s12602-026-10992-1.

## Highlights

Genome-informed comparative genomics confirmed the taxonomic identity of L. plantarum ND88.

ND88 showed 98% survival at pH 3 and high epithelial adhesion.

ND88 inhibited oral pathogens and reduced their adhesion to epithelial cells.

ND88 showed no hemolysis, low D-lactate production, and no acquired antibiotic resistance genes.

This study illustrates a genome-informed approach for identifying candidate probiotic strains.

**Supplementary Information:**

The online version contains supplementary material available at 10.1007/s12602-026-10992-1.

## Introduction

Lactic acid bacteria (LAB) have long been recognized for their beneficial roles in food fermentation and human health. Beyond their utility in improving food preservation and sensory properties, LAB are widely used in functional foods, dietary supplements, and clinical settings due to their probiotic properties, including modulation of gut microbiota, enhancement of gut barrier function, and regulation of immune responses [10, 31, 55, 67]. In recent years, growing evidence has also highlighted the potential of probiotics in broader applications such as oral health, metabolic regulation, and neuroimmune modulation [11, 12, 56].

With the advancement of molecular phylogenetics, the traditional genus *Lactobacillus* has undergone major taxonomic reclassification, resulting in the division into 23 novel genera and one emended genus to better reflect evolutionary relationships among strains [85]. For instance, *Lactobacillus plantarum* has been reclassified into *Lactiplantibacillus*, while *L. casei* and *L. rhamnosus* now belong to *Lacticaseibacillus*, and *L. reuteri* and *L. brevis* are classified as *Limosilactobacillus* and *Levilactobacillus*, respectively. These revisions provide a more refined framework for understanding strain-specific functionality and facilitate the development of targeted probiotic strategies.

Although LAB are generally regarded as safe, safety cannot be assumed at the species level and must instead be evaluated at the strain level. Some strains may possess undesirable features such as hemolytic activity, production of harmful metabolites like bile salt hydrolase (BSH) or D-lactate, or the presence of transferable antibiotic resistance genes. Major international authorities such as FAO/WHO, EFSA, and FDA have issued guidelines emphasizing that all probiotic strains intended for human use must undergo rigorous safety assessment, including both phenotypic analyses and genomic characterization [1], Araya, et al., 2002;

Our research group has previously conducted comprehensive safety assessments on various Bifidobacterium strains, including *B. bifidum* BGN4*, B. longum* BORI, and *B. animalis* subsp. *lactis* AD011, highlighting the importance of strain-specific evaluations and demonstrating that strains from the same species can exhibit significantly different functional traits [41, 43]. Currently, systematic studies on newly isolated LAB strains with potential applications in both the gastrointestinal and oral environments remain scarce, and research on the physiological characteristics of such strains (e.g., acid tolerance, intestinal adhesion) and their antimicrobial effects against oral pathogens is even more limited.

To address these gaps, recent advances in genome-informed analysis have provided powerful tools for predicting probiotic functionality prior to wet-lab validation. genome-informed analysis trained on large-scale Pfam and COG domain datasets can infer key probiotic traits, including adhesion, stress resistance, antimicrobial potential, and carbohydrate utilization. In this study, we integrated a genome-informed domain-level functional prediction model to analyze the ND88 genome. The model highlighted genomic signatures associated with gastrointestinal survivability and mucosal adhesion, which helped prioritize the phenotypic assays and informed the functional interpretation of ND88's potential capabilities.

In this study, we conducted a comprehensive safety and functional evaluation of *L. plantarum* ND88, a newly isolated strain with promising dual-target probiotic potential. Guided by the genome-informed insights, the experimental validation focused on verifying predicted traits such as acid tolerance, epithelial adhesion, and antimicrobial activity. We assessed the safety profile of ND88 through analyses of hemolytic activity, BSH activity, D-lactate production, and antibiotic susceptibility, complemented by whole-genome sequencing to screen for antibiotic resistance genes. To determine its gastrointestinal viability, we examined ND88’s survival under low pH and its adhesion to human intestinal epithelial cells. In addition, we explored its potential application in oral health by evaluating its ability to inhibit major oral pathogens and to reduce their adhesion to oral epithelial cells. Through this integrated approach, the present study aims to characterize the safety, physiological properties, and oral antimicrobial activity of ND88, thereby establishing a foundation for further evaluation of its functional potential.

## Materials and Methods

### Isolation of Microorganism

Fecal samples were collected from healthy adult volunteers recruited at Kangwon National University Hospital (IRB No. KNUH-2020–07–013). The samples were stored at –80 °C in stool collection tubes until use. For bacterial enrichment, the fecal samples were incubated in *Bifidobacterium* selective broth (KisanBio, Seoul, Republic of Korea, Cat. #MB-B0727) at 37 °C for 16 h. The cultured suspensions were then streaked onto TOS-MUP (KisanBio, Seoul, Republic of Korea, Cat. #MB-T0892) agar plates and incubated at 37 °C for 48 h. Although TOS-MUP is widely used as a selective medium for Bifidobacterium species, certain lactobacilli are known to grow under these conditions. Colonies with distinct morphologies were preliminarily selected and subsequently streaked onto MRS agar (BD DifcoTM, Sparks, MD, USA, Cat. #288,210) supplemented with 0.05% L-cysteine (Sigma-Aldrich, St Louis, MO, USA, Cat. #C7880) for final isolation of pure strains. The taxonomic identification of the isolated strain was performed using 16S rRNA gene sequencing and whole-genome analysis.

### Whole Genome Sequencing and Functional Gene Detection

Genomic DNA from strain ND88 was sequenced using the PacBio Sequel platform (HiFi reads) at CJ Bioscience. Long-read sequencing libraries were prepared according to the manufacturer’s protocol. Raw reads were assembled de novo using the SMRT Link v10.1.0 Microbial_Assembly_HiFi workflow and further polished with Unicycler v0.4.9. Genome completeness and contamination were evaluated using BUSCO and CheckM. Detailed sequencing statistics, assembly parameters, quality-control procedures, and additional comparative genomic analyses including average nucleotide identity (ANI) calculations are provided in the Supplementary Methods.

#### Genome Annotation and ORF Prediction

Structural gene prediction and functional annotation were performed using the CJ Bioscience in-house prokaryotic genome annotation pipeline. Coding sequences were predicted using Prodigal v2.6.2, and RNA genes were identified using tRNAscan-SE and Infernal. Functional annotation was conducted against curated protein databases (Swiss-Prot, KEGG, SEED, EggNOG) using USEARCH v8 and BLAST-based similarity searches. Full details of software versions and parameters are described in Supplementary Methods**.**

#### Pfam-Based Domain Annotation and Functional Classification

Pfam-based domain annotation was performed using HMMER (hmmscan, v3.4) against the Pfam-A HMM database (InterPro, Pfam v38.0) using trusted cutoffs [6, 19, 54]. For comparative analysis, protein sequences predicted from ND88 and 20 publicly available *L. plantarum* reference genomes retrieved from GenBank were subjected to the same Pfam domain annotation workflow to enable consistent cross-strain comparison. Domain hits were parsed and summarized with custom R scripts, and functionally categorized into probiotic-relevant classes (adhesion, transport, metabolism, regulation, others). Additional details of filtering criteria, DIAMOND-based validation [8] and visualization procedures are provided in Supplementary Methods.

#### Pangenome Analysis

For pangenome inference, ND88 and 20 publicly available *L. plantarum* genomes were re-annotated using a consistent Prokka pipeline to minimize annotation bias (Supplementary Table S1). Pangenome clustering was performed using Panaroo (v1.5.1) with strict cleaning settings. Core clusters were defined as those present in all genomes (21/21; ≥ 99%), and a near-core threshold (≥ 20/21; ≥ 95%) was additionally reported for sensitivity. ND88-specific orthogroups were defined as clusters present only in ND88, whereas ND88-enriched orthogroups were defined by increased copy number in ND88 relative to the strain set (copy number estimated from the number of gene identifiers reported per cluster). To support identification of candidate surface/adhesion loci beyond product-name annotations, ND88 proteins were screened for LPxTG motifs and for signal peptide–like N-termini together with C-terminal transmembrane–like segments; these sequence features were used as evidence tags when summarizing ND88-specific/enriched loci.

#### Probiotic Potential Prediction using MetaProbiotics

The assembled ND88 genome (FASTA format) was analyzed using metaProbiotics (https://github.com/zhenchengfang/metaProbiotics), a genome-informed analysis tool for assessing probiotic potential. Analyses were run on a Linux virtual machine (Oracle VirtualBox). The program outputs a tab-delimited file containing the FASTA filename, the single-bin probiotic score (0–1), and the final classification result. According to the default criteria, genomes with probiotic scores > 0.5 were classified as probiotic, whereas those with scores ≤ 0.5 were classified as non-probiotic.

#### Possible Toxigenic Gene Detection

Possible toxigenic genes were assessed by in silico screening of the whole-genome sequence using the VirulenceFinder 1.5 server hosted by the Centre for Genomic Epidemiology (CGE) [35]. The analysis was performed against curated virulence gene databases targeting known toxin- and enterotoxin-associated genes from major pathogenic bacteria, including *Escherichia coli*, *Enterococcus*, *Listeria*, and *Staphylococcus aureus*. BLAST-based searches were conducted with a sequence identity threshold of 90% and a minimum coverage of 60%.

#### Prophage Prediction Analysis

Putative prophage regions in the genome of strain ND88 were screened in silico using the PHASTER web server with default parameters. Predicted prophage regions were classified based on PHASTER completeness scores provided by the tool.

#### Horizontal Gene Transfer Assessment

Genome-based assessment of horizontal gene transfer (HGT) potential was conducted through integrated in silico analyses. Genome annotation was performed using Prokka (v1.14.5), and mobile genetic element–associated genes were identified based on annotation screening and manual curation. Detailed procedures are described in the Supplementary Methods.

#### Antibiotic Resistance Gene Analysis

To identify antibiotic resistance genes, the assembled genome sequence in FASTA format was uploaded to the ResFinder 4.5.0 web tool, which screens for acquired antimicrobial resistance genes by comparing the sequence to a curated database with BLAST algorithms [7, 25]. All results were interpreted according to European Food Safety Authority (EFSA) guidelines to ensure proper evaluation of antimicrobial resistance for food safety assessment (EFSA Panel & Feed, 2012; R. EFSA Panel, Guido, et al., 2018).

### Safety Assessment of ND88

#### Antibiotic Resistance Test

To evaluate the antibiotic resistance profile of ND88, the Minimum Inhibitory Concentration (MIC) was determined using E-TEST strips (BioMérieux) [45], US FDA K210757). The antibiotics used in this assessment were clindamycin (Catalog No. 412315), kanamycin (412382), tetracycline (412471), ampicillin (412253), erythromycin (412334), gentamicin (412368), and chloramphenicol (412309).

Antimicrobial susceptibility was determined using ETEST strips according to the FDA 510(k) decision summary (K210757). ND88 was cultured in MRS broth and adjusted to the appropriate turbidity, then uniformly inoculated onto MRS agar plates. After application of ETEST strips, plates were incubated at 37 °C, and MIC values were read at the point where the inhibition ellipse intersected the strip scale.

#### Hemolytic Property Test

Evaluation of hemolytic degradation activity of ND88 was carried out based on the previous protocol [43]. *Listeria ivanovii* subsp. *ivanovii* KCTC 3444 was used as a positive control strain for hemolytic degradation activity. ND88 and positive control strains were cultured in blood agar based on de Man, Rogosa and Sharpe (MRS) (BD Difco™, Franklin Lakes, NJ, USA) supplemented with 5% sheep blood (KisanBio, Seoul, Republic of Korea) at 37℃ for 48 h. After anaerobic cultivation, strains showing no color change was classified as non-hemolytic (γ -hemolytic). In contrast, strains showing clear zones due to red blood cell lysis were considered hemolytic (β-hemolytic) bacteria and a strain with a greenish halo appeared around the colonies were classified partially hemolytic (α-hemolytic) [43].

#### D-Lactate Production

To quantify D-lactate production by ND88, the strain was cultured in MRS broth (BD Difco™, USA) at 37 °C following a two-step incubation procedure [28]. Briefly, 1% (v/v) of thawed stock was pre-cultured for 15 h, and subsequently sub-cultured (1%, v/v) into fresh MRS for 24 h. Cultures were centrifuged (12,000 rpm, 1 min), and cell-free supernatants were collected. Uninoculated MRS incubated under identical conditions served as the negative control.

D-lactate concentration was determined using a colorimetric D-Lactate Assay Kit (Abcam, ab83429, Cambridge, UK) according to the manufacturer’s protocol. Absorbance at 450 nm was measured after 30 min incubation, and concentrations were calculated by subtracting background absorbance and interpolating values from a standard calibration curve [73].

#### Bile Salt Hydrolase Activity

BSH activity was evaluated using MRS agar supplemented with 0.05% (w/v) L-cysteine and 0.5% (w/v) taurodeoxycholic acid (TDCA), as previously described [69]. BSH activity was determined based on the formation of an opaque white precipitation halo surrounding colonies, resulting from bile salt deconjugation and subsequent deoxycholic acid precipitation. Strains were classified as BSH-positive when a clearly visible precipitation zone was observed after 48 h incubation, whereas strains lacking visible halo formation were considered BSH-negative.

*Bifidobacterium animalis* subsp. *animalis* ATCC 25527 was included as a positive control. Overnight cultures of ND88 and the control strain were spotted (10 µL) onto BSH agar plates and incubated anaerobically at 37 °C for 48 h prior to visual assessment.

### Functional Characterization of ND88

#### Adherence to HT-29 Cells

The adherence capacity of probiotic ND88 strain was assessed following a modified method by [16]. At first the probiotic strain ND88 is first pre-cultured by inoculating 1% (v/v) into MRS broth supplemented with 0.05% L-cysteine and incubated for 18–24 h. For further activation, 1% (v/v) of the pre-culture is transferred to fresh MRS broth containing 0.05% L-cysteine and incubated under the same conditions. After incubation, the culture is centrifuged at 4,000 rpm for 10 min. The resulting cell pellet is washed twice with PBS and then resuspended in DMEM for serial dilution to prepare the test solutions (according to specified concentration and dilution factor). To determine the initial viable cell count (M₀), 1 mL from the appropriate serial dilution is plated on MRS agar and incubated at 37 °C for 48 h. Simultaneously, HT-29 cells are seeded in 24-well plates using DMEM and cultured in a 5% CO₂ incubator at 37 °C until a monolayer form (typically 3–5 days). After removing the culture medium, the prepared probiotic test antibiotic-free medium is added to the wells and incubated for 90 min at 37 °C in a 5% CO₂ atmosphere. Following incubation, the wells are washed twice with PBS to eliminate non-adherent probiotic cells. Adherent probiotic cells are detached using trypsin–EDTA, diluted, and plated on MRS agar. After 48 h of incubation at 37 °C, the number of adherent viable cells (M₁) is determined. The adhesion efficiency is calculated as the ratio of adherent cells (M₁) to the initial viable cells (M₀).

#### Assessment of Acidic Tolerance

Survival under acidic conditions is a critical criterion for evaluating the functionality of probiotics, as they must endure the low pH environment of gastric juice following oral administration. A modified method by Behbahani et al. [5] was employed to test the acid tolerance of the probiotic strain ND88 [5]. To assess the acid tolerance of ND88, viable cell counts were measured after exposure to acidic media. The strain was activated and then inoculated at 10% (v/v) into MRS broth adjusted to pH 2.0, pH 3.0, pH 4.0, pH 5.0 and pH 6.5, using 1 N HCl. After incubation at 37 °C for 2 h, the cultures were serially diluted in buffered phosphate saline and plated on MRS agar to determine viable counts.

#### Inhibitory Activity against Oral Pathogens

To evaluate the inhibitory activity of ND88 against oral pathogens, co-culture assays were performed as previously described with minor modifications [37, 82]. For *Streptococcus mutans* KCTC 3065, a 1:1 mixture of MRS broth (supplemented with 0.05% L-cysteine) and BHI broth was used. For *Fusobacterium nucleatum* KCTC 2640, a 1:1 mixture of MRS(0.05% L-cysteine) and RCM broth was applied. For *Porphyromonas gingivalis* KCTC 5352 and *Prevotella intermedia* KCTC 15693, MRS broth (0.05% L-cysteine) was mixed 1:1 with KCOM1 broth. ND88 and each pathogen were co-inoculated at an initial concentration of 1 × 10⁷ CFU/mL per strain to standardize starting cell density. Pathogen-only cultures in the same mixed media served as controls.

Co-cultures were incubated anaerobically at 37 °C for 24 h (n = 3). After incubation, total genomic DNA was extracted from 1 mL of culture using the MagMax Microbiome Ultra kit. Species-specific bacterial abundance was quantified by RT-qPCR using previously validated primers (Supplementary Table S2). Standard curves generated from serially diluted genomic DNA corresponding to known CFU counts were used to convert Ct values into absolute cell numbers. Growth inhibition was determined by comparing final pathogen abundance in co-culture relative to the pathogen-only control under identical conditions.

To investigate the genetic basis of the observed antimicrobial phenotype, genome mining of the ND88 assembly was performed using antiSMASH and BAGEL4 to identify secondary metabolite and bacteriocin-associated gene clusters (see Supplementary Methods for detailed workflow and parameters).

#### Inhibition of Pathogen Adhesion to Oral Epithelial Cells

The inhibitory effect of ND88 on pathogen adhesion to oral epithelial cells was evaluated using KB cells, as previously described with minor modifications [17]. KB cells were maintained in RPMI 1640 supplemented with 10% fetal bovine serum and antibiotics at 37 °C in 5% CO₂. Cells were seeded in 24-well plates (3.0 × 10^5^ cells/well) and grown to confluence.

ND88 and each oral pathogen (*S. mutans*, *F. nucleatum*, and *P. intermedia*) were prepared at 1.0 × 10⁸ CFU/mL. For the adhesion inhibition assay, ND88 and each pathogen were mixed at a 1:1 ratio in antibiotic-free medium and applied to KB monolayers for 1 h at 37 °C. Pathogen-only treatment under identical conditions served as the positive control. After incubation, wells were washed with PBS to remove non-adherent bacteria, and adherent cells with attached bacteria were detached using trypsin–EDTA.

Adherent pathogens were quantified by species-specific qPCR as described above (Sect. 2.4.3). Adhesion inhibition was determined by comparing pathogen abundance in the co-treatment group relative to the pathogen-only control.

The authors used ChatGPT (OpenAI) and Perplexity as programming assistants during development of bioinformatic scripts. All generated code was reviewed, tested, and validated by the authors before use.

## Results and Discussion

### Genetic and Functional Characterization

**Fig. 1:**
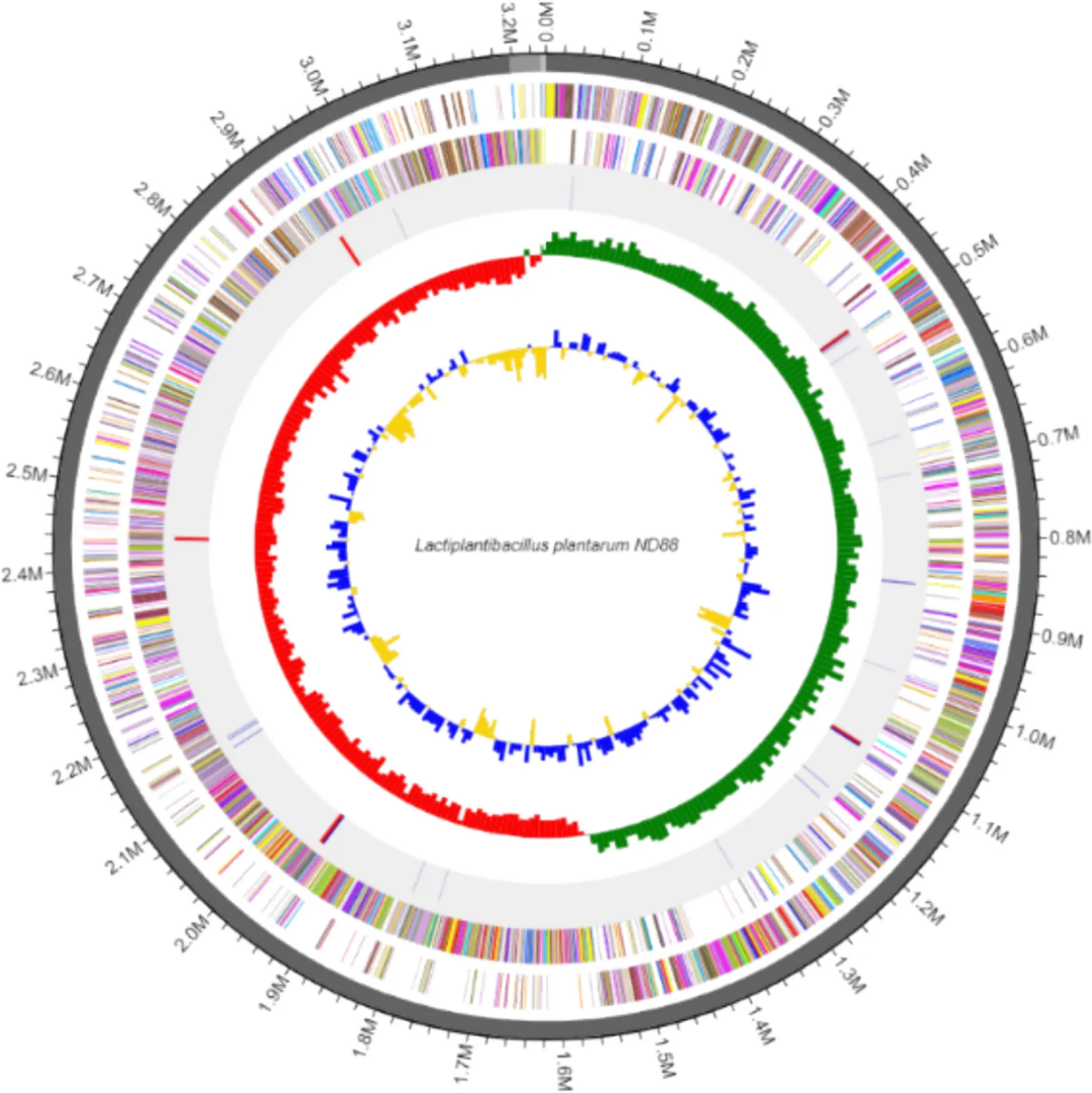
Circular genome map of ND88.The outermost circle represents the complete genome (3,236,305 bp). The second and third circles display coding sequences (CDSs) on the forward and reverse strands, respectively, color-coded according to Clusters of Orthologous Groups (COG) classifications. The fourth circle shows RNA genes, where blue and red bars indicate tRNA and rRNA genes, respectively. The fifth circle presents the GC skew, with green and red peaks representing positive and negative skew. The innermost circle illustrates the GC content, where blue and yellow peaks indicate high and low GC ratios, respectively. (G: Guanine, C: Cytosine; CDS: Coding DNA sequence; COG: Clusters of Orthologous Groups)

We obtained whole-genome sequencing data using the PacBio Sequel platform (Sequel_10K chemistry). The final assembly generated a genome of 3,236,305 bp in length with a GC content of 44.52%. The genome was assembled into three contigs with an N50 value of 3,197,760 bp and an average sequencing coverage of 100 × using SMRT Link v10.1.0 followed by Unicycler v0.4.9 for assembly refinement. No plasmids were detected in the assembly. Genome annotation predicted 3,009 coding sequences (CDSs), 16 rRNA genes, and 68 tRNA genes. The genomic map and COG-based functional classification of *L. plantarum* ND88 are shown in Fig. 1 and Table 1. A BLAST analysis of the 16S rRNA gene obtained from the whole-genome sequence revealed 100% identity (1489/1489 bp) with the 16S rRNA gene of *L. plantarum* ATCC 14917, confirming the taxonomic designation of the strain as ND88.

**Table 1:**
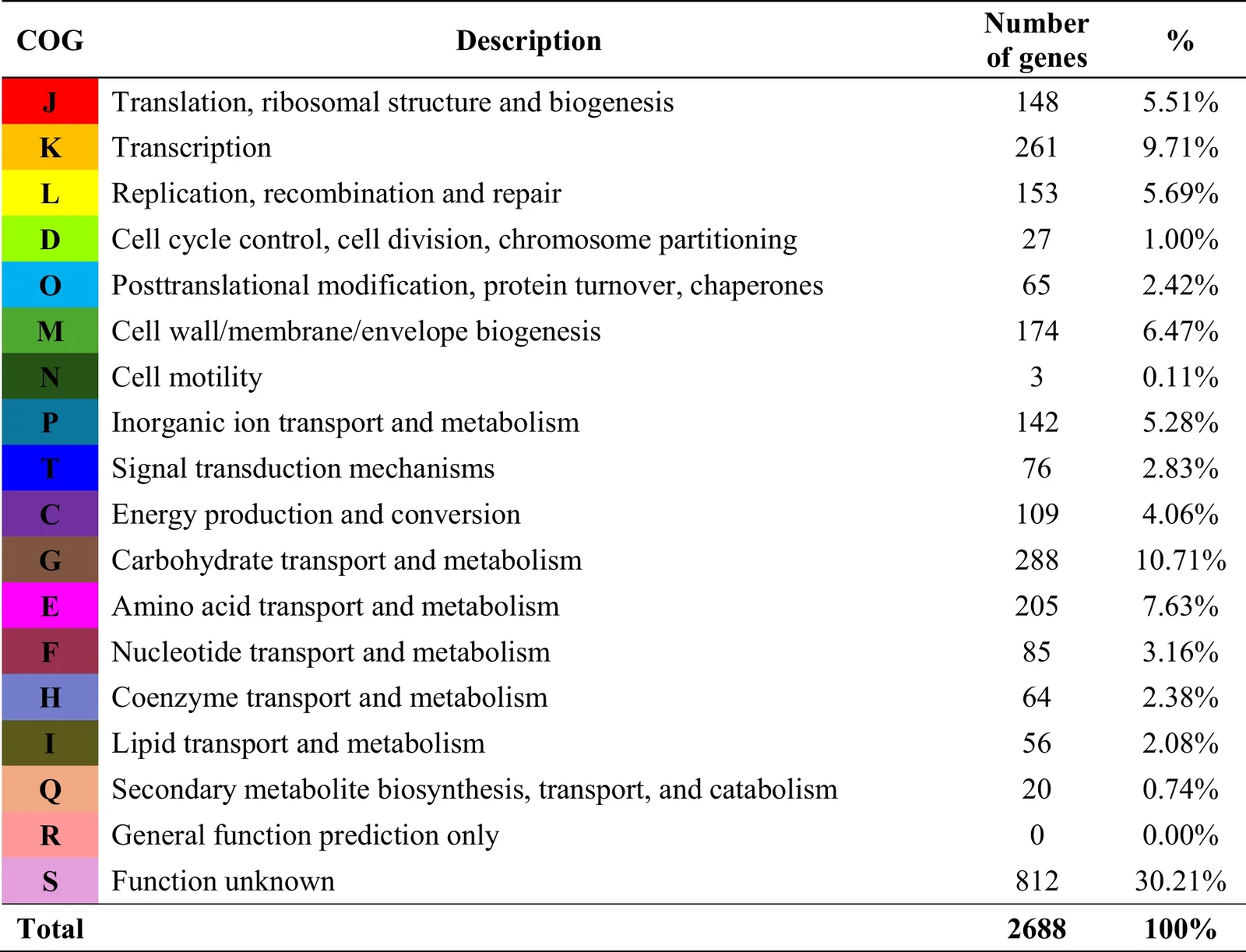
COG-based functional distribution of ND88 genes. The table shows the number and percentage of genes assigned to each Cluster of Orthologous Groups (COG) functional category in the ND88 strain. Each colored bar corresponds to a major functional group, such as translation (J), transcription (K), metabolism (G, E, F, H, I), signaling (T), cellular processes (D, N, M, O), and others. The chart provides insight into the physiological and metabolic capabilities of ND88, highlighting its genetic diversity in transport, metabolism, and regulatory functions

To further validate the taxonomic position, whole-genome average nucleotide identity (ANI) analysis was performed against 24 reference strains, including the type strains of *L. plantarum*, *L.paraplantarum* and *L.pentosus* (Supplementary Table S1andFigure S1). ND88 showed very high genomic similarity to all *L. plantarum* reference genomes, with ANI values ranging from 98.73% to 99.07% and 935–1,016 of 1,076 fragments mapping between ND88 and each reference. In contrast, ANI values between ND88 and *L. paraplantarum* strains were approximately 87.8%, and between ND88 and *L. pentosus* strains approximately 82.8%, well below the commonly accepted prokaryotic species boundary of about 95–96% ANI. These results robustly place ND88 within the *L. plantarum* species cluster and clearly distinguish it from the closely related species *L. paraplantarum* and *L. pentosus.*

Genome quality assessment further demonstrated high assembly completeness and negligible contamination. BUSCO analysis using the bacteria_odb10 dataset identified 99.2% complete BUSCOs (98.4% single-copy and 0.8% duplicated), with 0% fragmented and 0.8% missing BUSCOs. Consistently, CheckM analysis based on the marker lineage *o__Lactobacillales* estimated 98.77% completeness with 0.00% contamination and 0.00% strain heterogeneity (Supplementary Table S3). These complementary metrics indicate that the ND88 genome assembly is highly complete and free of detectable contamination.

MetaProbiotics, a genome-informed screening tool that predicts probiotic potential from metagenomic binning data using a language-model algorithm, was employed to computationally assess whether ND88 could be classified as a probiotic strain. This platform extracts genomic features from metagenome-assembled genome (MAG) bins, converts them into language-model embeddings through natural language processing, and classifies each bin using a random forest model trained on verified probiotic datasets (Wu, et al., 2024). Such in silico analysis provides a rapid and cost-effective approach to pre-screen strains with putative probiotic-associated traits prior to experimental validation. The metaProbiotics analysis predicted ND88 as a probiotic, suggesting that its genomic composition shares similar patterns with those of verified probiotic strains. In the present study, ND88 demonstrated inhibitory effects against oral pathogenic bacteria in vitro, which supports its potential role in maintaining a balanced oral microbiota. The genomic prediction therefore provides additional support for the probiotic potential of ND88. However, since the metaProbiotics model was primarily trained on human gut microbiota, its prediction accuracy may vary for strains from other ecological niches. Thus, the computational classification of ND88 as a probiotic should be regarded as supportive evidence rather than definitive proof, and further in vivo and clinical studies are required to confirm its efficacy and safety (Wu, et al., 2024).

Although metaProbiotics was originally trained on large-scale human gut microbiome datasets, its application in this study was considered appropriate given that ND88 is a human gut–derived *L. plantarum* isolate. At present, gut microbiome–based datasets remain the most comprehensive and systematically curated resources for probiotic trait prediction, and alternative models specifically trained on oral microbiome data are limited. Therefore, metaProbiotics was employed here as a hypothesis-generating tool rather than a definitive predictor of probiotic function.

Nevertheless, we acknowledge that probiotic traits relevant to the oral cavity are shaped by ecological pressures distinct from those in the gastrointestinal tract, and the applicability of gut-trained models for predicting oral probiotic potential has not been formally validated. Accordingly, the predicted probiotic scores should be interpreted with caution, particularly in the context of oral health. In this study, assays evaluating the inhibition of oral pathogens represent an initial, exploratory step toward assessing potential oral benefits. Future work will extend these analyses to investigate ND88’s functional roles within the gastrointestinal environment, thereby aligning experimental validation more directly with the model’s training domain and enabling a more comprehensive evaluation of strain-specific probiotic potential.

Although artificial intelligence–based tools were employed in this study, the primary novelty does not reside in the AI model itself. Instead, this work demonstrates a genome-informed, strain-specific characterization of ND88, in which in silico predictions were integrated with whole-genome safety assessment and experimental validation of oral-pathogen inhibitory activity. While oral probiotic functions and *L. plantarum* species have been widely reported, functionally validated evidence remains highly strain dependent. Our findings highlight how genome-guided screening can support the identification and prioritization of candidate strains, while experimental assays remain essential for confirming strain-specific phenotypes.

COG classification of the genome revealed that the five most abundant categories were "Function unknown" (30.21%), "Carbohydrate transport and metabolism" (10.71%), "Transcription" (9.71%), "Amino acid transport and metabolism" (7.63%), and "Cell wall/membrane/envelope biogenesis" (6.47%). This result is similar to what is commonly observed in other bacterial genomes, where metabolic, regulatory, and structural functions are dominant [44]. The proportion of genes with unknown function was relatively high at 30%, suggesting the presence of many uncharacterized or potentially novel genes, and indicating the need for further functional studies.

#### Comparative Pfam Domain Profiling of ND88 and Reference *Lactiplantibacillus plantarum* Genomes

**Fig. 2:**
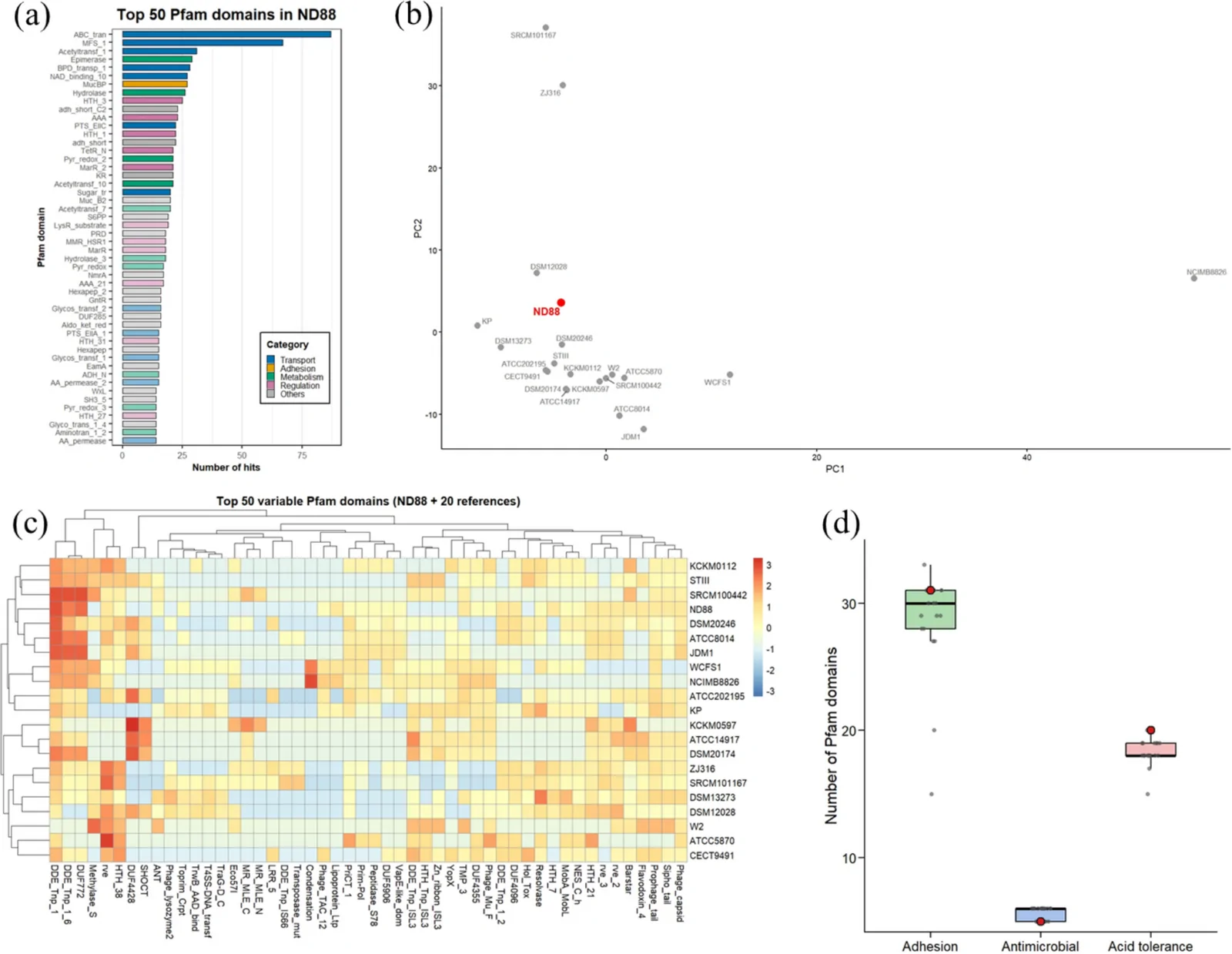
**Comparative Pfam domain profiling of *Lactiplantibacillus plantarum* ND88 and reference genomes.** (a) Distribution of major Pfam domains identified in the ND88 genome based on Pfam database annotation. Bars represent the frequency of major protein domain categories detected in the ND88 proteome (b) Principal component analysis (PCA) of log-transformed Pfam domain counts across 21 *L. plantarum* genomes. Most strains formed a coherent cluster, with ND88 positioned within the main cluster rather than as an outlier. (c) Heatmap showing the 50 most variable Pfam domains across all strains based on row-scaled z-scores. ND88 clusters with 20 reference strains, indicating broadly comparable domain composition. (d) Comparative distribution of Pfam domain counts across three functional categories among the 21 *L. plantarum* genomes, highlighting moderate variability among strains.

A total of 5032 Pfam domains were identified across the ND88 proteome using the Pfam-A database (HMMER, v3.4; trusted cut-off). The identified domains encompassed a wide range of functional categories, including transport, metabolism, regulation, and adhesion (Fig. 2a). Transport-associated families such as ABC_tran and MFS_1 were predominant, reflecting the strain’s extensive capacity for nutrient uptake and efflux of toxic compounds. Domains related to metabolic processes (Acetyltransf, Hydrolase, Epimerase) and transcrip-tional regulation (HTH, MarR, LysR) were also abundant, suggesting broad metabolic versa-tility and a responsive regulatory network that may facilitate environmental adaptation with-in the gastrointestinal tract (Surve, Shinde and Kulkarni, 2022).

Adhesion-related Pfam domains were highly represented, including MucBP (mucin-binding protein), LysM (lysin motif), and sortase. These domains are classically associated with bacterial attachment to epithelial or mucosal surfaces, which is an essential trait for probiotic colonization, persistence, and competitive exclusion of pathogens. (Du et al., 2022). In total, 18 open reading frames (ORFs) were predicted to encode adhesion-related proteins. Domain-architecture analysis further showed that many of these proteins contained multiple MucBP or LysM repeats co-localized with sortase motifs, reinforcing their potential for stable cell-wall anchoring and host interaction (Fig. 2a).

Among all annotated Pfam domains, MucBP was the most abundant, followed by LysM and MucBP_2, underscoring the strain’s strong mucosal binding potential. Additional domains such as CBAH (choloylglycine hydrolase family; putative bile salt hydrolase), ATP-synthase subunits, and pyridoxal decarboxylase were also identified, corresponding to energy metabolism and amino-acid modification. Together, these features indicate that ND88 possesses a well-balanced probiotic genomic architecture supported by adhesion capability, metabolic versatility, and regulatory robustness (Salvetti, Elisa and O’Toole, 2017).

To assess functional similarity between ND88 and established *L. plantarum* strains, Pfam domain profiles were compared across ND88 and 20 reference genomes using a uniform annotation pipeline (Supplementary Table S1).

Principal component analysis (PCA) of log-transformed Pfam domain counts showed that most strains formed a coherent cluster (Fig. 2b) ND88 grouped within this primary cluster rather than forming a distinct outlier, indicating that its overall functional domain composition is consistent with typical *L. plantarum* strains. In contrast, NCIMB8826 and ZJ316/SRCM101167 exhibited greater separation along principal components, reflecting broader inter-strain variability.

A heatmap of the 50 most variable Pfam domains across all strains (row-scaled z scores) revealed that ND88 clustered with several reference strains (Fig. 2c). However, localized increases in domains related to phage and mobile genetic elements were observed.

Comparative analysis of 21 *L. plantarum* genomes revealed moderate variability in Pfam domain counts across the three functional categories (Fig. 2d). In adhesion-related domains, variation was largely driven by differences in MucBP copy number. ND88 possessed 27 MucBP domains along with collagen-, fibronectin-, and sortase-associated domains, placing it within the upper range of the strain distribution. This pattern suggests that ND88 maintains a domain repertoire comparable to strains previously reported to possess host interaction–related surface proteins.

Acid tolerance-related domains exhibited moderate inter-strain variation. ND88 contained multiple heat shock proteins (HSP70, HSP20, and HSP33), Na⁺/H⁺ antiporters, and E1–E2 ATPases, resulting in a combined acid tolerance domain count located in the upper range of the overall distribution. This profile suggests robust stress-response capacity consistent with gastrointestinal adaptation.

Antimicrobial-associated domains, represented primarily by EntA_Immun, showed limited variability across strains, with most genomes harboring five to six copies. ND88 contained five copies, positioning it within the main distribution range and indicating conservation of bacteriocin immunity-associated elements comparable to other *L. plantarum* strains.

Overall, ND88 consistently fell within the upper distribution range across adhesion, antimicrobial, and acid tolerance categories. Rather than representing an extreme outlier, ND88 displayed a functionally enriched yet typical *L. plantarum* Pfam domain architecture, supporting its classification as a genomically representative strain within the species.

#### Integrative Functional Prediction from Pfam and COG Profiles

Pfam annotation of ND88 revealed that transport-related domains constituted a disproportionately large fraction of the top-ranked families, with multiple copies of ABC_tran, MFS_1, BPD_transp_1, AA_permease, and Sugar_tr. This pattern is consistent with comparative genomics of *L. plantarum*, where expansion of ABC and major facilitator superfamily (MFS) transporters has been linked to bile tolerance, multidrug efflux, and broad nutrient uptake capacity [79]. The high prevalence of transporter domains in ND88 therefore suggests an enhanced ability to import diverse carbohydrates, amino acids, and inorganic ions and to extrude bile salts, organic acids, antibiotics, and other toxic compounds, supporting improved survival during GI passage and a competitive advantage in nutrient-limited niches.

ND88 also carries multiple adhesion-related domains, notably mucin-binding (MucBP) repeats. MucBP- and Mub-like surface proteins are key determinants of mucus adhesion in *lactobacilli* and have been implicated in stable colonization of the intestinal mucosa [74]. In the well-characterized probiotic strain *Lactocaseibacillus rhamnosus* GG(LGG), a mucus-binding factor and pilus-associated mucus-binding proteins confer strong adherence to human intestinal mucus and epithelial cells [38]. Consistent with these observations, the repeated MucBP domains and enriched COG category M in ND88 indicate a strong capacity for mucosal adhesion and persistence, potentially contributing to competitive exclusion of pathogens and sustained production of beneficial metabolites [47].

In addition to transport and adhesion functions, ND88 encodes a diverse repertoire of transcriptional regulators, including multiple helix–turn–helix (HTH_1/HTH_3) families and members of the MarR and TetR regulator families. Comparative regulatory network reconstruction in lactic acid bacteria has shown that such regulators orchestrate transcriptional responses to carbohydrate availability, oxidative stress, bile, and antimicrobial compounds [63]. The relatively high proportion of COG category K (transcription) observed in ND88 mirrors previous *L. plantarum* genome surveys, which emphasize the species’ extensive regulatory capacity as a basis for its “nomadic” lifestyle and adaptation to diverse habitats [53]. These regulatory features suggest that ND88 can dynamically adjust gene expression in response to pH, oxygen, osmolarity, and nutrient fluctuations in the GI tract.

Metabolism-related Pfam domains in ND88 are also broadly distributed, with abundant acetyltransferase, epimerase, and hydrolase families, in line with the large metabolic gene complement typical of *L. plantarum* [53]. COG analysis shows substantial representation of categories G (carbohydrate transport and metabolism), E (amino acid transport and metabolism), and C (energy production and conversion), comparable to other *L. plantarum* strains that display marked metabolic versatility and the capacity to utilize a wide range of dietary substrates [53]. This metabolic breadth suggests that ND88 can utilize diverse dietary carbohydrates and amino acids, enabling stable growth and metabolite production under fluctuating nutrient conditions. Strains with similar metabolic repertoires have been shown to modulate short-chain fatty acid levels and improve metabolic and inflammatory markers in vivo, suggesting that ND88 may share these beneficial properties [26].

In the present study, genome-informed analysis was applied as a hypothesis-generating tool to provide genomic-level interpretations of experimentally observed adhesion phenotypes. Rather than directly validating the function of individual predicted adhesion-related proteins, this approach aimed to identify candidate domains, such as MucBP, that may plausibly contribute to the observed strain-level adhesion behavior. This phenotype-first, genome-informed framework allows for mechanistic inference while avoiding overinterpretation of predictive genomic features.

Overall, the combination of (i) an expanded set of nutrient and efflux transporters, (ii) MucBP-rich adhesion modules and cell-envelope biogenesis genes, (iii) a dense network of transcriptional regulators involved in stress responses, and (iv) a broad metabolic enzyme repertoire constitutes a genomic “probiotic signature” that is characteristic of versatile and robust *L. plantarum* strains [47]. Although experimental validation remains essential, the integrated Pfam–COG features suggest that ND88 harbors genomic signatures commonly associated with probiotic-related functions, including predicted tolerance to gastrointestinal conditions, putative mucosal interaction capacity, and metabolic versatility. To verify these genome-based predictions, we next evaluated the safety profile (Sect. 3.2) and experimentally assessed the functional properties of ND88, including acid tolerance, epithelial adhesion, and antimicrobial activity (Sect. 3.3).

#### Comparative Pangenome Analysis Identifies ND88-Specific Orthogroups

Pangenome analysis of 21 *L. plantarum* genomes identified 5,093 gene clusters, including 2,401 core clusters present in all strains (21/21) and 2,692 accessory clusters variably distributed across genomes. Using a relaxed presence threshold (≥ 20/21 strains), 2,492 clusters were retained as the near-core genome.

Within this framework, ND88 contained 69 strain-specific orthogroups absent from all comparator genomes, and 15 ND88-enriched orthogroups defined by increased copy number relative to the strain set. Because product-name annotations alone were often non-specific (e.g., hypothetical proteins), we additionally integrated sequence-based evidence for surface localization. Screening the ND88 proteome identified 18 LPxTG-motif proteins, and cross-referencing with the ND88-specific/enriched orthogroups showed that 13 of 84 candidates carried surface-associated sequence features (LPxTG and/or signal peptide plus C-terminal transmembrane signatures) (Supplementary Table S4).

The pangenome structure observed for the 21 *L. plantarum genomes,* consisting of a substantial accessory fraction in addition to the core genome, is consistent with the well-recognized genomic plasticity of this species. Previous comparative studies have demonstrated that *L. plantarum* possesses an open pangenome in which many traits associated with environmental adaptation and probiotic function are located within the accessory genome rather than the core genome [81]. In this context, the identification of 69 ND88-specific orthogroups and 15 ND88-enriched orthogroups supports the presence of strain-level genomic variation that may contribute to phenotypic differences relative to comparator strains.

Among the phenotypes evaluated in this study, the pangenome results are most directly linked to adhesion. Surface-associated proteins are widely recognized as key mediators of interactions between *lactobacilli* and host-associated surfaces, including mucus and epithelial cells. LPxTG-containing proteins are particularly important because they are covalently anchored to the cell wall by sortase and have been implicated in adhesion-related phenotypes in *L. plantarum* [62]. Consistent with this framework, ND88 encodes 18 LPxTG-motif proteins, and 13 of the ND88-specific or enriched candidates contain LPxTG motifs or other sequence features consistent with surface localization. Although these observations do not demonstrate that each candidate directly mediates adhesion, they suggest that ND88 carries an expanded repertoire of cell-envelope-associated proteins that are mechanistically compatible with the strong adhesion phenotype observed in vitro.

Functional annotation based on Pfam and COG analyses further supports this interpretation. Adhesion-related Pfam domains were frequently detected, including MucBP, LysM, and sortase-associated domains. These protein domains are classically associated with bacterial attachment to epithelial or mucosal surfaces and are considered important for probiotic colonization, persistence, and competitive exclusion of pathogens (Du et al., 2022). In total, 18 ORFs were predicted to encode adhesion-related proteins, and several exhibited domain architectures containing multiple MucBP or LysM repeats in combination with sortase-recognition motifs, suggesting potential for stable cell-wall anchoring and host interaction.

More broadly, the Pfam and COG functional profiles indicate that ND88 possesses genomic features commonly associated with probiotic functionality in *L. plantarum*. These include an expanded repertoire of nutrient transporters and efflux systems, genes related to cell-envelope biogenesis and adhesion modules, multiple transcriptional regulators involved in environmental stress responses, and a diverse metabolic enzyme complement. Together, these genomic characteristics resemble the functional architecture previously described for adaptable and metabolically versatile *L. plantarum* strains [47]. While experimental validation will be required to confirm the specific roles of individual loci, the integrated genome-based evidence suggests that ND88 harbors genomic signatures consistent with probiotic-related traits, including potential tolerance to host-associated environments, mucosal interaction capacity, and metabolic versatility.

In contrast, the pangenome summary provides less direct insight into antimicrobial activity. In *L. plantarum*, antimicrobial function is typically mediated by discrete bacteriocin biosynthetic loci rather than broad enrichment of general functional domains. As a result, bacteriocin-related genes may not be readily apparent in high-level orthogroup summaries. Previous studies have successfully used genome mining tools such as BAGEL4 to identify plantaricin clusters in *L. plantarum* [33]. Therefore, identification of a BAGEL4-predicted plantaricin locus in ND88 would provide a more specific genomic explanation for the observed antimicrobial phenotype than orthogroup-level analysis alone.

Acid tolerance–related domains exhibited moderate inter-strain variation across the analyzed genomes. ND88 contained multiple stress-response–associated domains, including heat shock proteins (HSP70, HSP20, and HSP33), Na⁺/H⁺ antiporters, and E1–E2 ATPases, resulting in a combined acid tolerance–associated domain count located in the upper range of the overall distribution. These elements are commonly involved in maintaining intracellular pH homeostasis and protecting cellular proteins under acidic stress, suggesting that ND88 possesses a stress-response repertoire compatible with survival in low-pH environments. However, the present pangenome analysis does not yet provide a direct genomic explanation for acid tolerance. Acid resistance in lactic acid bacteria is typically associated with systems such as F₁F₀-ATPase, amino acid decarboxylation pathways, malolactic fermentation, and broader stress-response networks [51]. Because the ND88-specific and enriched orthogroups identified in this study were prioritized primarily based on surface-localization features, they are more readily interpreted in relation to adhesion rather than acid adaptation. These results suggest that the acid tolerance phenotype observed in vitro likely reflects the presence of conserved stress-response systems rather than strain-specific genomic innovations. Further gene-level analysis will be required to clarify the precise genetic basis of this phenotype.

Taken together, these results support a cautious interpretation that ND88 exhibits strain-level genomic variation that plausibly contributes to its adhesion phenotype through an expanded repertoire of surface-associated proteins. Antimicrobial activity appears more appropriately explained by targeted bacteriocin-associated loci, whereas acid tolerance remains supported by phenotypic evidence but is not yet directly linked to the present pangenome analysis.

### Safety Assessment of ND88

#### Antibiotic Resistance Genes and Phenotypic Resistance Profiles

The antibiotic resistance of ND88 was evaluated using both phenotypic and genotypic approaches. Phenotypic susceptibility testing was performed according to EFSA guidelines (EFSA Panel & Feed, 2012; EFSA Panel, Guido, et al., 2018), and the minimum inhibitory concentrations (MICs) of seven antimicrobial agents, such as ampicillin (sodium salt), gentamicin (sulfate), kanamycin, tetracycline, erythromycin, chloramphenicol, and clindamycin (hydrochloride), were determined [71]. ND88 showed MIC values above the EFSA microbiological cut-off values for gentamicin (32 mg/L) and kanamycin (> 256 mg/L), whereas all other tested antibiotics exhibited MIC values below the recommended thresholds.Although vancomycin and streptomycin are typically included in lactic acid bacteria assessments, they were excluded in this study, as they are not required for *L. plantarum* according to current guidelines (Table 2).

**Table 2:** Minimum inhibitory concentrations of antimicrobial agents for ND88 based on EFSA cut-off values (EFSA Panel & Feed, 2012). The MIC values of ND88 were compared with the EFSA-defined microbiological cut-off values for *L. plantarum*. Values exceeding the cut-off suggest potential resistance

Antimicrobial agent	*L. plantarum* (cut-off value) (mg/L)	*L. plantarum* ND88 (mg/L)
Ampicillin (sodium salt)	2	0.032
Gentamicin (sulfate)	16	32
Kanamycin	64	> 256*
Tetracycline	32	24
Erythromycin	1	1.0
Chloramphenicol	8	6
Clindamycin (hydrochloride)	2	1.0

* > indicates that the MIC value exceeded the highest concentration tested

Additional evidence from the study by Tuerhong et al. [75] indicates that the antibiotic resistance patterns of *L. plantarum* are highly conserved among commercial and wild-type isolates, further supporting that ND88 exhibits a typical resistance profile for its species and does not harbor concerning outlier values.

Overall, the data demonstrates that the antibiotic resistance characteristics of ND88 are within the normal range previously reported for *L. plantarum* species, satisfying international safety standards for use in food and probiotic applications. Nevertheless, ongoing genome-based monitoring is recommended, especially for resistance markers not yet included in current reference databases.

To explore whether the elevated MIC values to gentamicin and kanamycin in ND88 may be associated with acquired resistance determinants, the web-based tool ResFinder 4.5.0 was used in accordance with EFSA guidelines. The whole-genome sequencing FASTA file of ND88 was uploaded as the input for the analysis. The results showed that no genes associated with gentamicin and kanamycin resistance were identified in the genome of ND88 (Supplementary Figure S2). The whole-genome sequencing FASTA file of ND88 was uploaded as input, applying detection thresholds of ≥ 90% sequence identity and ≥ 60% minimum length. The ResFinder output explicitly reported “No gene sequences found in resistant genes” under the “Acquired AMR gene hits” section (Supplementary Figure S2), indicating that no known acquired gentamicin- or kanamycin-associated resistance genes were detected in the ND88 genome under the applied criteria. Although elevated MIC values to kanamycin and gentamicin have been reported in certain *L. plantarum* strains, accumulating evidence suggests that aminoglycoside resistance in this species is predominantly intrinsic rather than acquired [14]. Aminoglycosides require active transport across the cytoplasmic membrane, a process that depends on an oxygen-dependent electron transport chain. As a facultatively heterofermentative lactic acid bacterium lacking a functional respiratory chain under standard growth conditions, *L. plantarum* exhibits reduced aminoglycoside uptake, resulting in naturally elevated MIC values.

Consistent with this mechanism, large-scale surveys of *Lactobacillaceae* have demonstrated high proportions of field isolates classified as resistant to kanamycin and, to a lesser extent, gentamicin, despite the absence of corresponding acquired resistance determinants in their genomes [14]. Importantly, EFSA distinguishes between intrinsic (chromosomally encoded, non-transferable) resistance and acquired, potentially transferable resistance. In addition, genome assembly did not identify any plasmid sequences, reducing the likelihood that aminoglycoside resistance determinants are located on transferable replicons.

Taken together, the absence of identifiable aminoglycoside resistance genes in ResFinder and the lack of plasmid sequences support the interpretation that the observed phenotype is more consistent with intrinsic reduced susceptibility rather than acquired resistance. Nevertheless, additional guideline-based MIC confirmation and transferability-focused investigations would further strengthen antimicrobial safety assessment.

#### In Silico Screening for Toxigenic Genes

To evaluate the potential presence of toxigenic determinants, the whole-genome sequence of ND88 was analyzed using the VirulenceFinder 1.5 server (Centre for Genomic Epidemiology, CGE). BLAST-based searches were performed against curated virulence gene databases targeting toxin- and enterotoxin-associated genes from major pathogenic genera, including Escherichia coli, Enterococcus, Listeria, and Staphylococcus aureus. The analysis applied a minimum sequence identity threshold of 90% and a minimum coverage of 60%.

No virulence-associated genes were detected in any of the evaluated categories. Specifically, no homologs of shiga toxin genes (*E. coli*), cytolysins or aggregation substances (*Enterococcus*), listeriolysin (*Listeria*), or staphylococcal enterotoxins, exoenzymes, host immune evasion factors, or toxin-related genes (*S. aureus*) were identified. All searches returned “No hit found” under the predefined criteria (Supplementary Figure S3).

From a safety assessment perspective, the absence of detectable toxin-encoding genes supports the genomic safety profile of ND88. The exclusion of known acquired virulence determinants is an important consideration in the evaluation of candidate probiotic strains. These findings indicate that ND88 does not harbor known toxigenic genes identifiable by sequence homology under the applied parameters.

Nevertheless, it should be acknowledged that in silico analyses are dependent on available databases and defined similarity thresholds. While no known virulence genes were detected in this study, continued updates of reference databases and complementary phenotypic safety assessments further strengthen the overall safety evaluation.

#### Prophage-Associated Regions

PHASTER analysis identified a total of four prophage-associated regions in the ND88 genome, including two intact and two incomplete regions (Supplementary Table S5). The presence of multiple prophage regions is consistent with previous reports indicating that *L. plantarum* frequently harbors prophages [60, 61]. Importantly, no genes annotated as known virulence factors or acquired antibiotic resistance determinants were detected within these prophage-associated loci based on our genome-wide safety screening. Although intact prophages may theoretically retain inducibility and act as vehicles for horizontal gene transfer, the presence of prophages per se is not considered a safety concern. Instead, safety assessments primarily focus on whether prophage-associated regions harbor transferable genes related to virulence or acquired antibiotic resistance. In ND88, no such safety–critical genes were identified within the predicted prophage regions. We acknowledge that in silico prediction alone cannot determine prophage inducibility; therefore, future studies involving experimental induction (e.g., mitomycin C–mediated prophage induction and characterization of released phage particles) will be necessary to directly evaluate inducibility and potential contributions to horizontal gene transfer.

#### Horizontal Gene Transfer Potential

In addition to the prophage-associated regions described above, the mobilome composition of ND88 was examined to assess its potential for horizontal gene transfer (HGT) (Supplementary Table S6). Genome annotation identified 15 insertion sequence (IS) family transposases, comprising nine IS1182 family members, three IS3 family members, and three other IS/Tn3-related transposases. Several tyrosine recombinases and integrases were also detected, including five XerC, one XerD, one XerH, and one prophage-like integrase.

Despite the presence of these mobile element-associated proteins, no genes encoding conjugation machinery such as relaxases or type IV secretion system components were identified. Although 15 genes annotated with the term “plasmid” were detected based on functional annotation, no plasmid replicon sequences or clear conjugative plasmid structures were identified. Furthermore, genome-wide screening did not detect any acquired antibiotic resistance genes.

Collectively, these findings indicate that ND88 contains a limited repertoire of mobile genetic element-associated genes but lacks key determinants required for active conjugative transfer. The absence of detectable conjugation machinery and acquired resistance determinants suggests a relatively low apparent capacity for horizontal dissemination of resistance or virulence traits under the conditions assessed. However, as these conclusions are based on in silico analysis, the inducibility of prophage regions and the actual frequency of potential mobilization events cannot be determined without experimental validation.

#### Hemolytic Properties and Metabolic Traits

**Fig. 3:**
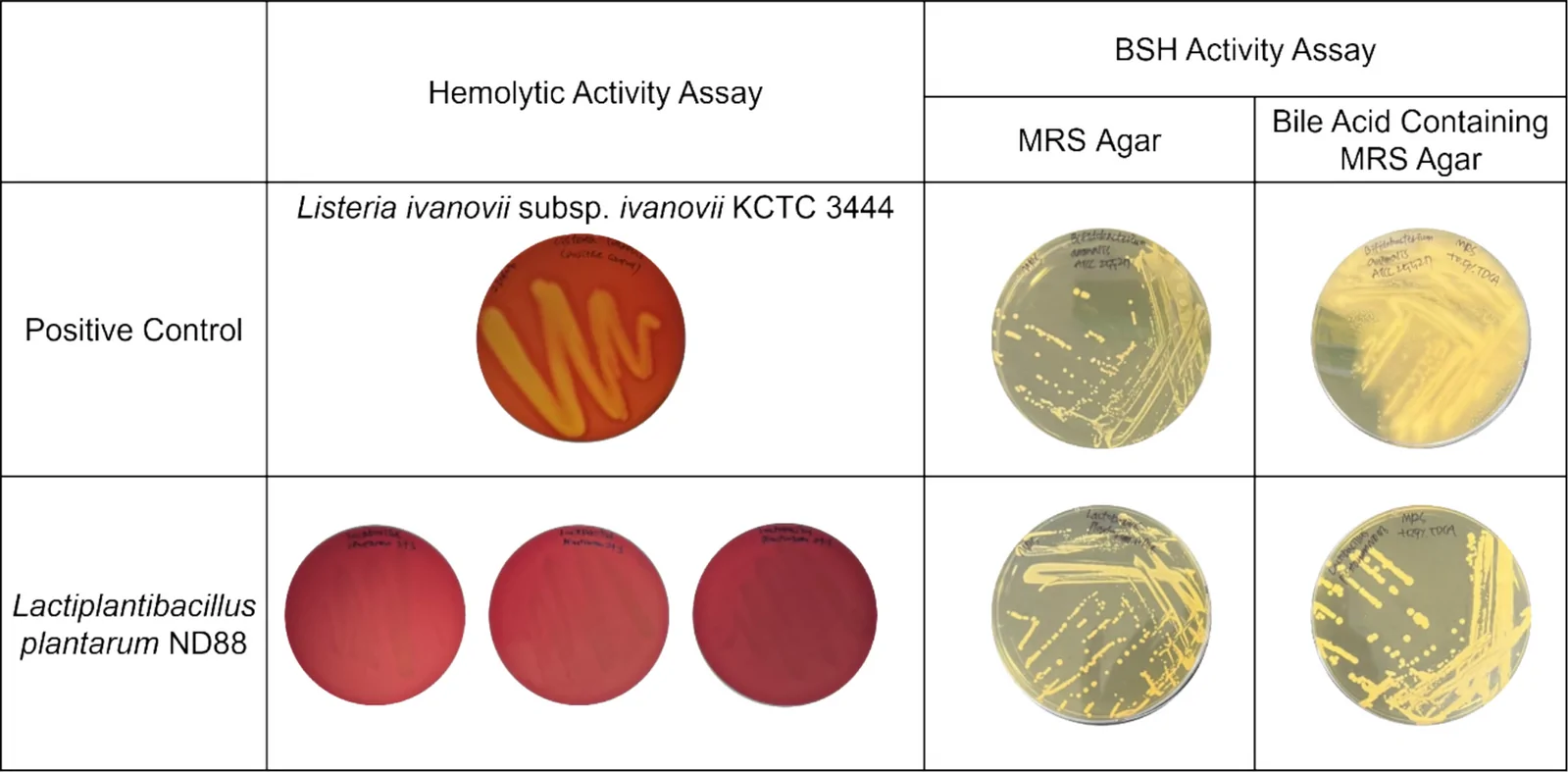
Hemolytic and BSH activities of ND88 compared to positive controls (*Listeria ivanovii* subsp. *ivanovii* KCTC 3444 for hemolytic activity, and *Bifidobacterium an imalis* subsp. *animalis* ATCC 25527 for BSH activity)

The safety of probiotic microorganisms within the host environment is a fundamental prerequisite for their application, and potential risks like red blood cell lysis. In line with this, authoritative guidelines form the (FAO/WHO, 2002) emphasize the importance of assessing hemolytic activity as a key criterion for probiotic safety. *L. plantarum* is mostly reported not to have hemolytic activities by various previous studies. *L. plantarum* TWK10 isolated from Pao-tsai and 25 out of 30 strains was non-hemolytic (γ-hemolysis strain) [24, 29]. However, confirming there is no hemolytic activity in the probiotic strain is crucial. In our hemolysis assay, *Listeria ivanovii* subsp. *ivanovii* KCTC 3444 served as the positive control and displayed transparent zones around colonies on blood agar plates, indicative of β-hemolysis (Fig. 3). In contrast, the colonies of ND88 did not produce visible clearing or discoloration zones on blood agar supplemented with 5% sheep blood under the tested conditions. These observations suggest that ND88 lacks detectable hemolytic activity and may be considered γ-hemolytic or non-hemolytic in phenotype. While further verification under varying conditions may be warranted, these results are consistent with established safety assessment criteria commonly applied to probiotic-related strains.

BSH is an enzyme that catalyzes the deconjugation of bile salts and is often considered beneficial for enhancing bile tolerance and regulating cholesterol levels [13]. However, the deconjugated bile salts can be further metabolized into secondary bile acids, such as deoxycholic acid (DCA), which have been implicated in carcinogenesis by suppressing anticancer T-cell immune responses [13]. Therefore, assessing BSH activity in probiotic strains is crucial.

Since *L. plantarum* is known to frequently exhibit BSH activity, we aimed to evaluate whether strain ND88 exhibits this enzymatic function [18]. ND88 did not exhibit detectable BSH activity under the assay conditions. No precipitation halo indicative of bile salt deconjugation was observed around colonies, in contrast to the positive control strain. Therefore, ND88 was classified as BSH-negative.

D-lactate is a safety concern in probiotic development because it can cause D-lactic acidosis, especially in infants with short bowel syndrome or surgically altered intestines. Some *Lactobacillaceae* species, including *L. plantarum*, can produce D-lactate. Therefore, probiotic strains should be tested for D-lactate production to ensure safety [64]. To evaluate the D-lactate production capacity of ND88, a D-lactate assay kit (Abcam, ab83429) was employed according to the manufacturer’s protocol. Uninoculated MRS broth was used as a negative control to account for background absorbance, which was subtracted from the raw measurements. Based on the corrected optical density values, ND88 was found to produce D-lactate at a final concentration of 0.00678 ± 0.00059 g/L. To assess whether the level of D-lactate production by ND88 is within an acceptable safety range, we compared its production level to that of strains previously granted GRAS (Generally Recognized As Safe) status. According to GRAS No. 953, *L. plantarum* strains KABP 011, 012, and 013 were reported to produce D-lactate at concentrations of 4.16 g/L, 3.93 g/L, and 3.82 g/L, respectively (Kaneka Americas Holding, 2020). ND88 produced only 0.00678 g/L of D-lactate under the same assay conditions indicating a substantially lower level. This comparison suggests that ND88 can be considered a safe strain with respect to D-lactate production.

### Functional Properties of ND88

#### Adhesive Ability to Intestinal Epithelial Cells

**Fig. 4:**
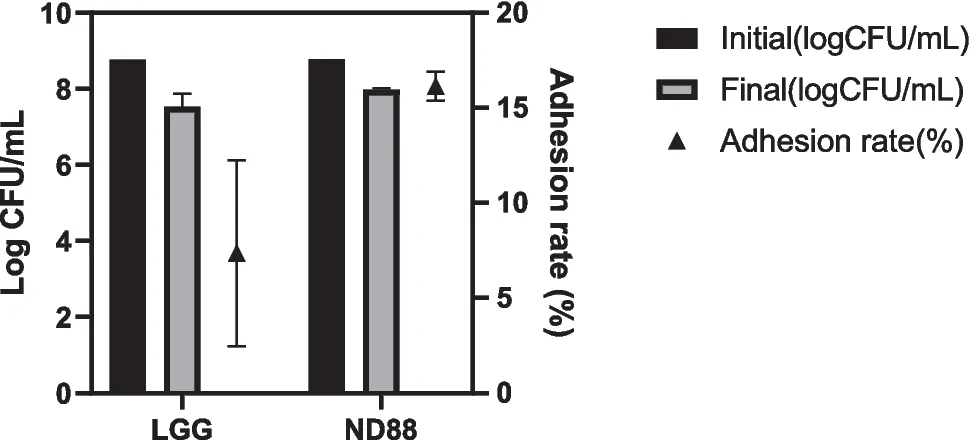
Adhesion ability of ND88 to intestinal epithelial cells compared with LGG. The figure illustrates the adhesion capacity of ND88 and the reference strain LGG to intestinal epithelial cells. The black and gray bars represent the viable cell counts (log CFU/mL) before and after adhesion, respectively, while the triangles indicate the adhesion rate (%). ND88 exhibited a comparable or slightly higher adhesion rate than LGG, suggesting its strong ability to attach to intestinal epithelial surfaces

Our results demonstrate that adhesion ability of probiotic strain ND88 to intestinal epithelial HT-29 cells was found to be significantly higher (16.15 ± 0.76%) than that of commonly used probiotic reference strain LGG, which demonstrated an adhesion level of 7.36 ± 4.88% (Fig. 4). This result indicates a high colonization potential of ND88 within the gastrointestinal tract. The adhesion capacity of LGG observed in our study is consistent with recent literature. For instance, Sharma et al. (2017) reported that LGG exhibited adhesion levels ranging from 5 to 12% on HT-29 and Caco-2 cells, varying with experimental conditions such as incubation time, pH, and mucin presence. Similarly, Zhang et al. (2022) noted that LGG showed moderate adhesion levels (approximately 6–10%) compared to novel isolates derived from fermented plant products, further confirming our observations [46].

Adhesion to the intestinal epithelium is considered a critical determinant of probiotic functionality, enabling persistence, competitive exclusion of pathogens, and localized immune modulation [27]. In this context, the strong adhesion potential of ND88 may be attributed to cell surface factors such as exopolysaccharides (EPS), surface-layer (S-layer) proteins, or lipoteichoic acids, which have been shown to mediate bacterial-host interactions (Y. [50]). Such adhesive potential of LAB probiotics also confirmed by Sagmeister et al. [68] elaborating the mechanism of probiotic function and immune modulation [68].

Furthermore, a study by Zhu et al. [86] highlighted that probiotic strain *L. plantarum* B2 with adhesion rates exceeding 22% demonstrated increased mucin-binding activity, leading to prolonged gastrointestinal residence time and enhanced delivery of bioactive compounds [86]. The adhesion level observed for ND88 places within the range reported for strains exhibiting strong in vitro adhesion, suggesting its relevance for further investigation of adhesion-associated functional traits.

The observed high adhesion capacity of ND88 provides a good ground for its development as a robust probiotic strain with potential applications in gut health modulation, inflammatory bowel disease management, and dietary supplement innovation in future.

The adhesion assay performed in this study was initially designed as a phenotypic evaluation of probiotic potential. The strong adhesion capacity of ND88 observed in this study may be related to the presence of multiple surface-associated proteins identified through genome-informed analyses. Pfam domain annotation and comparative pangenome analysis across 21 *L. plantarum* genomes revealed several proteins containing sequence features consistent with surface localization, including LPxTG motifs, signal peptides, and transmembrane regions. These genomic features are commonly associated with bacterial adhesion and host interaction in lactic acid bacteria.

However, comparative analysis also indicated that domains such as MucBP are not uniquely expanded in ND88 relative to other *L. plantarum* strains. Therefore, the present genome-informed analysis should be interpreted as providing genomic context for the observed adhesion phenotype rather than direct mechanistic validation of specific adhesion factors. Further functional studies would be required to determine the precise molecular determinants underlying the adhesion phenotype of ND88.

#### Survival of ND88 under Acidic Conditions

**Fig. 5:**
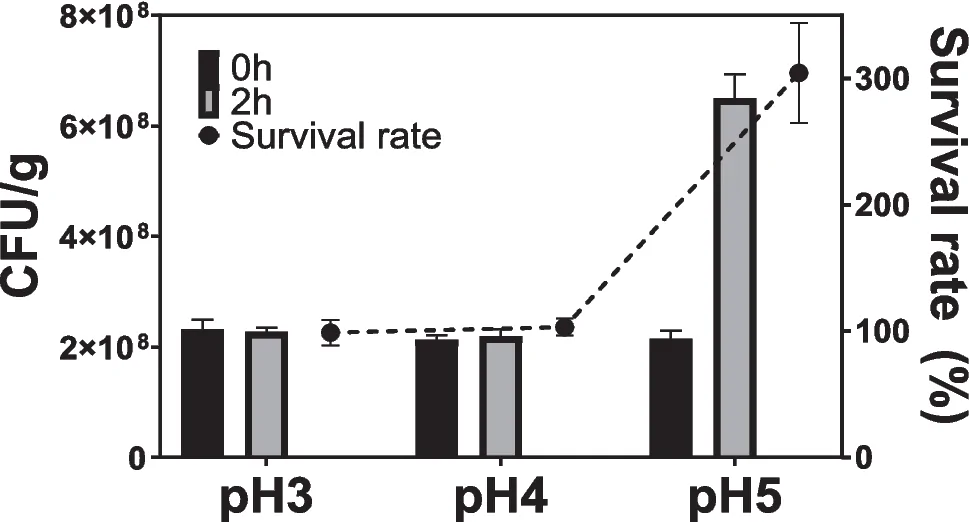
Acid tolerance of ND88 under different pH conditions. The figure shows the survival of ND88 after exposure to acidic environments (pH 3, 4, and 5) for 2 h. The black and grey bars represent viable cell counts (CFU/g) at 0 and 2 h, respectively, while the circles indicate the calculated survival rate (%). ND88 maintained stable viability at pH 3 and pH 4, while showing active growth at pH 5. Collectively, these findings suggest a degree of acid tolerance, including short-term survival under strongly acidic conditions (pH 3)

Viability of ND 88 under acidic condition over 2 h test at pH 3–4, remained essentially unchanged (2.0–2.3 × 10^8 CFU·g⁻^1^ at 0 h vs. 2 h), yielding 98**–**103% survival, which indicates that no meaningful loss. At pH 5 under mild oral-like acidity, cells increased from approximately 2.1 × 10^8 to 6.5–7.0 × 10^8 CFU·g⁻^1^ within 2 h. This corresponds to a ~ threefold increase, indicating net growth rather than mere persistence. Error bars were small relative to means, suggesting good repeatability (Fig. 5).

Our study that *L. plantarum* tolerates pH 3 for ≥ 2 h aligns with recent reports showing high strain-dependent survival in simulated gastric fluid. For example, GCC19M1 retained 93–97% viability at pH 3, and broader reviews cite approximately 44–113% survival ranges depending on strain, medium, and exposure time [75],Z. Zhang, et al., 2024). Consistently high survival has also been reported for strain N4, which exhibits approximately 87% viability in simulated gastric juice, and for strain HYY-DB9, which shows good tolerance throughout simulated gastrointestinal passage [15],Y.-h. [49]. However, it should be noted that the 2 h acid exposure applied in the present study represents a simplified and time-limited in vitro model of gastric transit and does not fully capture the dynamic and heterogeneous conditions of the human stomach. Accordingly, the observed survival at pH 3 should be interpreted as evidence of short-term acid adaptation rather than definitive proof of long-term or extreme acid resistance. In harsher pH 2 conditions that resemble the fasted stomach, survival drops markedly for some strains. For instance, *L. plantarum* AM2 shows only about 38% viability after 3 h, underscoring the importance of fed-state pH and residence time [48]. At pH 5, our observation of rapid population increase is compatible with reports that *L. plantarum* can remain highly viable and even proliferate under mildly acidic food/oral environments (fruit juices pH 3.4–3.7; oral niche), where counts often stay above 10^6 CFU·mL⁻^1^ for extended periods [30, 72]. Such growth at pH 5 suggests that once past the most acidic gastric window, *L. plantarum* can rebound quickly, particularly when matrix buffering and nutrients are available. Practically, these data indicate that the tested strain is gastric-tolerant at pH 3 (fed-state proxy) and thrives at pH 5 (mouth/upper-GI proxy). Taken together, these findings support a model in which ND88 exhibits transient tolerance and adaptive responses to acidic stress, enabling maintenance of viability during short-term exposure to gastric-like pH and subsequent expansion under milder acidic conditions, rather than uniform resistance across all gastric environments.

Mechanistically, this pattern is consistent with well-characterized acid-stress responses in *lactobacilli*, including activation of the proton-pumping F₁F₀-ATPase system, remodeling of membrane fatty-acid composition, and up-regulation of molecular chaperones. These coordinated responses help sustain viability at pH 3–4 and enable rapid recovery as environmental pH increases [9]. Our finding places the strain ND 88 among acid-tolerant *L. plantarum* capable of maintaining viability through gastric-like pH 3 and expanding at oral-like pH 5, in agreement with recent strain-level studies and reviews. Further testing at pH 2 and under dynamic digestion systems such as INFOGEST would help refine survival claims and strengthen the evidence base for application-specific formulations [77].

#### Inhibitory Activity of ND88 against Oral Pathogens

The modulation of the oral microbiome through probiotics represents a novel approach for addressing oral diseases associated with microbial dysbiosis [52]. We assessed the antagonistic properties of ND88 against four major oral pathogens. Co-culture experiments demonstrated that ND88 markedly inhibited the growth of all examined pathogens, suggesting its potential as an oral probiotic agent. Results are presented in Table 3.

**Table 3:** Inhibition Rates of Oral Pathogenic Bacteria by ND88 Treatment

Strains	Experimental Groups	Number of bacteria (cells/mL)	Inhibitory rate (%)
*S. mutans*	*S. mutans* KCTC3065	3.3 × 10^9^ ± 2.8 × 10^8^	
	*S. mutans* KCTC3065 + ND88	9.4 × 10^7^ ± 2.1 × 10^7 **^	97.1
*F. nucleatum*	*F. nucleatum* KCTC2640	2.0 × 10^9^ ± 4.1 × 10^7^	
	*F. nucleatum* KCTC2640 + ND88	1.0 × 10^9^ ± 2.3 × 10^8 *^	47.7
*P. gingivalis*	*P. gingivalis* KCTC5352	6.8 × 10^7^ ± 9.2 × 10^6^	
	*P. gingivalis* KCTC5352 + ND88	2.2 × 10^6^ ± 2.9 × 10^5 **^	96.7
*P. intermedia*	*P. intermedia* KCTC15693	4.3 × 10^8^ ± 1.7 × 10^8^	
	*P. intermedia* KCTC15693 + ND88	5.2 × 10^7^ ± 3.8 × 10^6^	87.8

Values are expressed as the mean ± standard deviation of three different experiments. (^*^p < 0.05, ^**^p < 0.01, ^***^p < 0.001).

The most profound inhibitory effects were observed against the primary cariogenic pathogen *Streptococcus mutans* (97.1% inhibition; p < 0.01) and the keystone periodontal pathogen *Porphyromonas gingivalis* (96.7% inhibition; p < 0.01). The effective inhibition of *S. mutans* is essential for caries management, probably facilitated by known mechanisms of ND88, including organic acid production that reduces environmental pH [30]. Similarly, the near-total inhibition of *P. gingivalis*, a key mediator of periodontal inflammation [58], indicates significant therapeutic potential. This effect is likely attributable to the secretion of antimicrobial compounds such as bacteriocins, which are produced by *L. plantarum* strains and demonstrate efficacy against pathogenic bacteria [3].

*Prevotella intermedia*, a bacterium closely linked to periodontitis (S. [83]), also exhibited a notable inhibition of 87.8%. In addition, the growth of *Fusobacterium nucleatum* was inhibited by 47.7% (p < 0.05). This effect, while moderate, holds significant relevance. *F. nucleatum* serves as a pivotal bridging organism that promotes the co-aggregation of both early and late colonizers in dental plaque; its suppression may hinder the maturation of pathogenic biofilms [59]. It should be acknowledged that the use of mixed culture media may influence bacterial growth due to differential adaptation of individual strains. Although baseline growth controls using the same mixed media were included to minimize this confounding effect, the contribution of medium-dependent factors cannot be completely excluded and should be considered when interpreting the inhibitory effects observed in co-culture.

ND88 exhibited inhibitory activity against representative oral pathogens, including *Streptococcus mutans*, *Fusobacterium nucleatum*, and *Prevotella intermedia*. The inhibition was reproducibly observed under the tested in vitro conditions. Organic acid production is a well-established antimicrobial mechanism of lactic acid bacteria and likely contributes to the observed effects, particularly under conditions permitting environmental acidification.

To explore whether additional antimicrobial determinants may be present, genome mining analysis was conducted. Bacteriocin-focused screening identified a plantaricin E–like class II bacteriocin gene cluster in the ND88 genome. The locus contains a predicted precursor peptide gene together with accessory genes typically involved in peptide processing and export [76]. Class II plantaricins produced by *L. plantarum* have been reported to exert antimicrobial activity primarily through membrane permeabilization in susceptible Gram-positive bacteria [34, 57].

In addition, secondary metabolite analysis detected a RiPP-like biosynthetic gene cluster. RiPPs (ribosomally synthesized and post-translationally modified peptides) are known to include diverse antimicrobial peptides [32, 65]; however, functional activity of this predicted cluster in ND88 has not been experimentally validated.

The presence of a plantaricin-type bacteriocin locus provides a plausible genomic basis for inhibition of *S. mutans*, a Gram-positive species that may be susceptible to membrane-active peptides. In contrast, inhibition of the Gram-negative species *F. nucleatum* and *P. intermedia* is more likely attributable to organic acid production and environmental pH reduction, which are established antimicrobial mechanisms of lactic acid bacteria [39, 66].

At present, the relative contribution of acid-dependent and peptide-mediated mechanisms cannot be distinguished based on the available data. Future experiments such as evaluation of antimicrobial activity using pH-neutralized cell-free supernatants and protease treatment assays would help clarify whether peptide-dependent activity contributes to the observed inhibition.

Overall, integration of genomic analysis with phenotypic observations indicates that ND88 harbors multiple potential antimicrobial determinants, although further functional validation is required to confirm their specific contributions. ND88 demonstrated inhibitory activity against major cariogenic and periodontopathic bacteria, including *S. mutans*, *P. gingivalis*, *P. intermedia*, and *F. nucleatum*. The variation in inhibition levels among species suggests that more than one antimicrobial mechanism may be involved. These findings support the potential of ND88 as a candidate for further investigation in the context of oral health applications.

#### Inhibition of Pathogen Adhesion to Oral Epithelial Cells by ND88

**Fig. 6:**
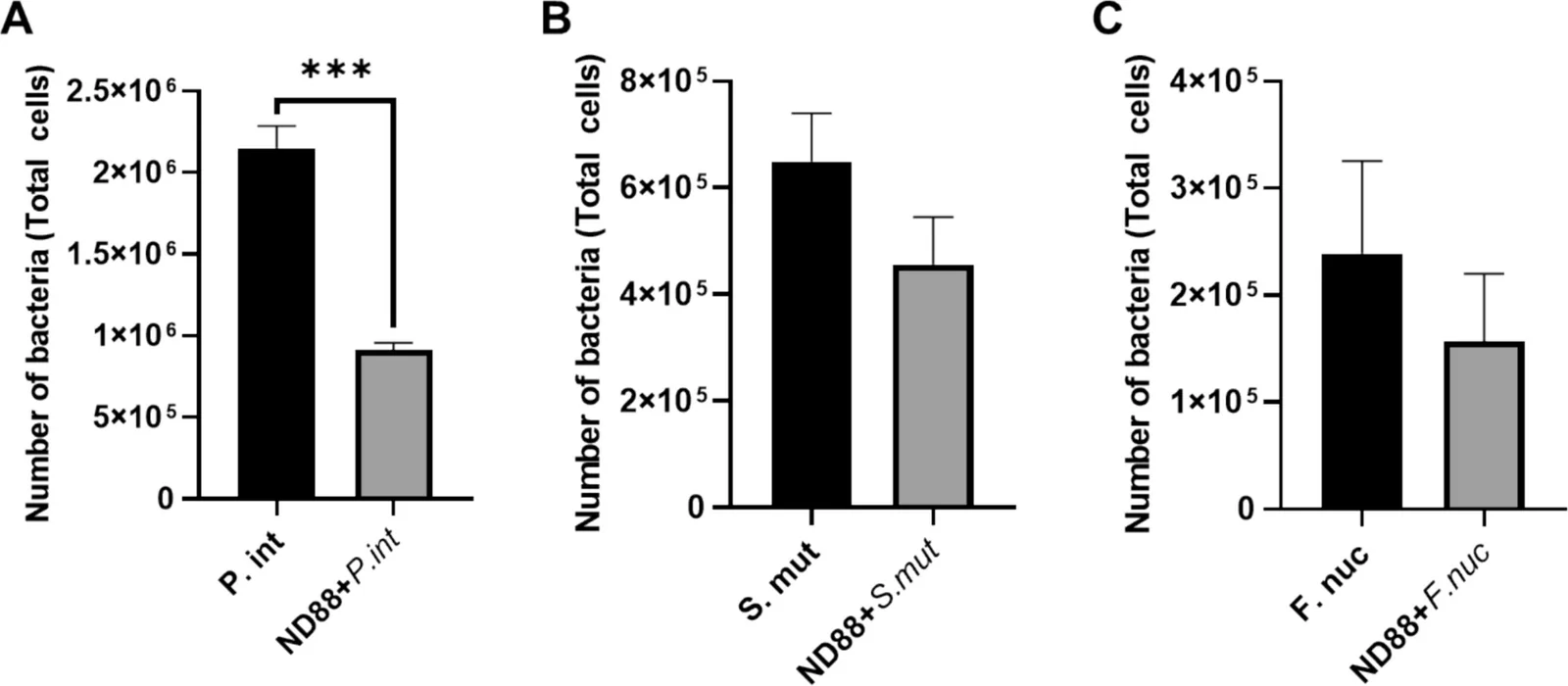
Inhibitory effects of ND88 on the initial adhesion of three major oral pathogens. *(P. intermedia* KCTC15693, *S. mutans* KCTC3065 and* F. nucleatum* KCTC2640)

Probiotics play a crucial role in oral health by preventing pathogenic colonization, which typically begins with the adhesion of bacteria to host epithelial surfaces [52]. This research assessed the capacity of ND88 to disrupt the initial stage of infection for three significant oral pathogens. The results presented in Fig. 6 indicate that ND88 demonstrates considerable anti-adhesion activity, implying an effective mechanism for influencing the oral microbiome and disease prevention.

The most significant inhibitory effect was observed against *P. intermedia* KCTC15693, with ND88 decreasing its adhesion to oral epithelial cells by 57.42%. *P. intermedia* is a gram-negative anaerobic bacterium significantly associated with the etiology of periodontal diseases (S. [83]). The attachment to the gingival epithelium is essential for colonization and subsequent tissue invasion, potentially eliciting a destructive host inflammatory response. The observed high rate of inhibition indicates that ND88 competes effectively with *P. intermedia*, presumably for specific receptors on the host cell surface. Competitive exclusion serves as a fundamental mechanism for probiotics; wherein beneficial bacteria inhibit the establishment of pathogens within the host environment [2]. ND88 demonstrates significant activity against a major periodontal pathogen, making it a promising candidate for promoting periodontal health.

Furthermore, the adhesion of *F. nucleatum* KCTC2640 was inhibited by 34.30%. This finding's significance is heightened by the distinct ecological role of *F. nucleatum*. *F. nucleatum* serves as a crucial intermediary that connects early colonizers to highly pathogenic late colonizers, playing a vital role in the structural development of complex biofilms [22]. Consequently, inhibiting its initial attachment can interfere with the overall architectural maturation linked to advanced periodontal disease.

The investigation also demonstrated a 29.82% inhibition of *S. mutans* KCTC3065, the principal causative agent of dental caries. The ability of *S. mutans* to adhere to tooth surfaces is a central mechanism of its pathogenicity, resulting in cariogenic plaque and acid production that leads to enamel demineralization [40].

Taken together, ND88 exhibits considerable potential as a targeted probiotic through its ability to competitively inhibit periodontal pathogens, including *P. intermedia*, disrupt the biofilm-scaffolding function of *F. nucleatum*, and reduce the colonization of *S. mutans*. These actions contribute to the maintenance of a healthy oral microbiome and proactively address the early stages of periodontal disease and dental caries [30].

## Conclusions

This study demonstrates the utility of integrating genome-based computational prediction with experimental validation for the systematic evaluation of strains with putative probiotic-associated traits. Pfam/COG analyses and MetaProbiotics-based predictions indicated that ND88 possesses key probiotic-associated features, including strong adhesion potential, metabolic versatility, and transporter-mediated stress tolerance. These genome-derived predictions were supported by experimental findings: ND88 exhibited a favorable safety profile, showed stable adhesion to epithelial cells, and demonstrated inhibitory and adhesion-blocking activity against oral pathogenic bacteria. Taken together, these results indicate that ND88 exhibits in vitro and genome-predicted features commonly associated with probiotic-related functions, including predicted tolerance to gastrointestinal conditions, putative mucosal interaction capacity, and pathogen-inhibitory activity. More broadly, this work highlights the value of combining genome-informed screening with targeted in vitro assays as an effective framework for accelerating the discovery and validation of novel functional probiotic strains.

## Supplementary Information

Below is the link to the electronic supplementary material.

Supplementary file1 (DOCX 652 KB)

## Data Availability

Data will be made available on request.
