# Supplementary material for: Genome-Informed Characterization of Lactiplantibacillus plantarum ND88 with Inhibitory Activity against Representative Oral Pathogens
Source: Probiotics Antimicrob Proteins. 2026 Mar 25;18(6):7858–79. doi: 10.1007/s12602-026-10992-1 (PMC13518469; doi:10.1007/s12602-026-10992-1)
Supplement: Supplementary file 1 [file 12602_2026_10992_MOESM1_ESM.docx]

**Supplementary Materials**

1. **Supplementary Method**

***DNA extraction and quality assessment***

Genomic DNA was extracted from Lactiplantibacillus plantarum ND88 using a standard microbial genomic DNA extraction protocol. DNA concentration was quantified using the PicoGreen dsDNA assay. Only samples with concentrations ≥150 ng/µL were selected for library preparation.

DNA integrity was initially evaluated by electrophoresis on a 1% agarose gel. Samples showing intact high-molecular-weight DNA without visible degradation were considered suitable for PacBio sequencing. When DNA quality was uncertain, additional electrophoresis was conducted using low-percentage agarose gels and extended run times to improve resolution of high-molecular-weight DNA fragments.

***PacBio library preparation and sequencing***

PacBio SMRTbell libraries were constructed according to the manufacturer’s recommended protocol for HiFi sequencing. Briefly, genomic DNA was mechanically sheared to an average fragment size of approximately 15–20 kb. DNA fragments underwent end-repair and ligation with SMRTbell adapters, followed by purification and size selection.

Sequencing was performed on the PacBio Sequel platform, generating highly accurate HiFi long reads suitable for microbial genome assembly.

***Genome Assembly and Polishing***

*De novo* assembly was conducted using the SMRT Link v10.1.0 Microbial_Assembly_HiFi protocol. Assembly polishing was performed using Unicycler v0.4.9 (unicycler_polish module). Polishing iterations were automatically executed in a convergence-based manner until no further base corrections were detected. Plasmid detection was performed during genome assembly, and no plasmids were identified.

***Genome Completeness and Contamination Assessment***

Genome quality was evaluated using BUSCO v6.0.0 with the bacteria_odb10 lineage dataset and CheckM v1.2.2 using the marker lineage *o__Lactobacillales*. Additional contamination screening was conducted using ContEst16S, and phylogenomic placement was confirmed using the UBCG pipeline.

***Average Nucleotide Identity (ANI)***

Whole‑genome ANI was calculated to confirm the species affiliation of strain ND88. ND88 was used as the query genome and compared against 20 *L*. *plantarum* reference genomes (including ATCC 8014, ATCC 14917, WCFS1, JDM1, ST‑III, NCIMB8826 and others, **Supplementary** **Table S1**) as well as type/representative strains of *L. paraplantarum* and *L. pentosus*. ANI was computed with FastANI v1.34 using default settings, with ND88 supplied as a single query file and all reference genomes listed in a text file (refs_all.txt). The command was of the form fastANI --query ND88.fna --refList refs_all.txt --threads 16 --output ND88_vs_all.fastani, and output columns (query, reference, ANI, matching fragments, total fragments) were parsed and harmonized by renaming ND88_contigs_renamed.fasta entries to ND88.

***Structural Gene Prediction***

Structural gene prediction was performed using the CJ Bioscience in-house prokaryotic genome annotation pipeline. Coding sequences were predicted using Prodigal v2.6.2 with parameters (-f gff -m -c -g 11). Transfer RNA genes were identified using tRNAscan-SE v1.3.1, and rRNA, tmRNA, and other non-coding RNAs were predicted using Infernal v1.1.1 against the Rfam 12.0 database. CRISPR loci were identified using PILER-CR v1.06 and CRT v1.2.

***Genome annotation and functional characterization***

Structural gene prediction and genome annotation were performed using the CJ Bioscience prokaryotic genome annotation pipeline. Coding sequences were predicted using Prodigal v2.6.2, while RNA genes were identified using tRNAscan-SE for tRNA detection and Infernal for non-coding RNA identification. Functional annotation of predicted proteins was carried out using USEARCH v8 (ublast mode) against curated protein databases including Swiss-Prot (151210), KEGG (20181001), SEED Subsystems (151210), and EggNOG v4.5, using parameters -evalue 1e-5, -threads 8, -accel 1.0, and -maxaccepts 1. Additional protein similarity searches and domain mapping were performed using NCBI BLAST+ v2.2.30+ with an e-value threshold of 1e-4.

***Pfam-based domain annotation***

Pfam-based domain annotation was performed using HMMER (hmmscan, v3.4) against the Pfam-A HMM database (InterPro, Pfam v38.0; Pfam Consortium, 2025) (Mistry et al., 2021; Eddy, 2011; Blum et al., 2021). The *--cut_tc* option was applied to retain only hits meeting Pfam trusted cutoff thresholds. The representative command was:

*hmmscan --cut_tc --domtblout ND88.all2.domtbl Pfam-A.hmm ND88_proteins.faa*

The generated *domtblout* file contained domain coordinates, E-values, and functional descriptions and was processed for downstream analyses.

***Processing and summarizing Pfam domain profiles***

All Pfam hits were parsed using custom Bash and R scripts. The workflow included: Extract-ing domain names, Counting total hits and unique domain families, Merging counts into summary tables (ND88_pfam_counts.txt), Functional categorization of domains (see below) and Visualization of top domains.

In total, 5,032 Pfam domain hits and ~1,900 unique domain occurrences were identified across the ND88 proteome. To ensure data quality, hits with incomplete coordinates, ex-tremely low model coverage, or truncated alignments were removed through custom filter-ing scripts.

***Functional categorization of Pfam domains***

Each Pfam domain was assigned to a curated probiotic-relevant functional class.

Classification was based on: Literature-based functional roles, Domain descriptions from Pfam / InterPro, Known motifs from probiotic genomes (*Lactobacillaceae*), Custom rules generated from the expanded ORF flagging workflow

Domains were grouped as follows:

Adhesion-related: MucBP (PF06458), MucBP_2 (PF17965), LysM (PF01476), Sortase (PF04203)

Transport: ABC_tran, MFS_1, BPD_transp_1, Sugar transporters

Metabolism: Epimerase, NAD_binding_10, Acetyltransf_1 and Pyr_redox families

Regulation: HTH_1, MarR and LysR_substrate

Others: Housekeeping, unknown, or non-probiotic-related domains

The final classification table (pfam_flagged_expanded.tsv) was generated using a hybrid manual + automated pipeline for accuracy.

To confirm and refine functional predictions, homology-based searches were performed against reference protein databases using DIAMOND blastp (<https://github.com/bbuchfink/diamond>) (Buchfink, Reuter, & Drost, 2021). Specific probiotic-related features (e.g., MucBP, LysM, Sortase) were identified based on alignment coverage and sequence identity thresh-olds following internal quality control criteria.

***Visualization of Pfam domain distributions***

All figures involving Pfam counts and functional categories were generated using RStudio (2025.05.1+513). Key libraries used: library(tidyverse), library(ggplot2), library(viridis). Figures generated include:Top 50 Pfam domains, Functional category bar plots, Color-coded do-main abundance charts, Custom layout with legend repositioning for publication. Plots were styled using custom themes, color palettes, and precise positioning to avoid over-lap with bars or domain tracks.8958

***Extraction and visualization of adhesion-related domain architectures***

Adhesion-related proteins were identified through keyword filtering in the Pfam domtbl file:

*grep -E 'MucBP|MucBP_2|LysM|Sortase' ND88.pfam_full.domtbl.*

For each protein, domain coordinates (start–end positions) were extracted and merged into a structural annotation table. Six representative proteins were selected based on: Large size (>200 aa), Presence of multiple repeated MucBP or LysM units, Presence/absence of Sortase anchoring motifs and Likely surface-exposed architecture typical of L. plantarum adhesion proteins. Domain architecture was plotted using custom R scripts with geom_rect and ge-om_segment, mapping domain blocks along the protein length.

***Pfam domain annotation and comparative analysis***

Pfam domain-based functional profiling was performed to compare ND88 with 20 publicly available *L. plantarum* reference strains (total n = 21). Protein domain annotations were generated using HMM-based searches against the Pfam database, and Pfam identifiers were standardized by removing version suffixes from domtblout outputs. Functional comparison focused on three biologically relevant categories associated with probiotic potential: adhesion-related domains (including MucBP, collagen-binding, fibronectin-binding, and sortase-associated domains), antimicrobial-related domains (primarily bacteriocin immunity-associated domains such as EntA_Immun), and acid tolerance-related domains (including HSP70/DnaK, HSP20, HSP33, Na⁺/H⁺ antiporters, and E1–E2 ATPases).

For each strain, domain counts were calculated per category, and comparative distributions across strains were visualized using violin plots. ND88 was highlighted within each distribution to assess its relative functional positioning among reference strains. This Pfam-based approach provides a standardized and widely validated framework for comparative genomic functional profiling without requiring full pangenome reconstruction.

***Pangenome clustering and orthogroup identification***

To enable comparative genomic analysis, ND88 and 20 publicly available *L*.*plantarum* genomes retrieved from GenBank were re-annotated using Prokka v.1.14.5 to standardize gene annotations across all genomes. Pangenome inference was conducted using Panaroo (v1.5.1) with strict cleaning parameters to minimize gene fragmentation and assembly-related artifacts. Gene clusters were classified as: Gene clusters were classified based on their presence across genomes. Core clusters were defined as those present in all genomes (21/21), and a near-core threshold (≥20/21) was additionally reported. ND88-specific orthogroups were defined as gene clusters present exclusively in ND88 and absent from all other genomes. ND88-enriched orthogroups were defined based on increased copy number in ND88 relative to the strain set. Copy number was estimated using the number of gene identifiers assigned to each cluster in the Panaroo gene presence–absence matrix.

***Screening of surface localization features***

ND88 protein sequences were screened for sequence features associated with surface localization, including LPxTG motifs characteristic of sortase-anchored proteins, signal peptide–like N-terminal sequences, and C-terminal transmembrane-like segments. These sequence features were used to annotate potential surface-associated proteins among ND88-specific or enriched orthogroups identified in the pangenome analysis.

***Genome mining of secondary metabolite and bacteriocin gene clusters***

To investigate the genetic basis of the antimicrobial phenotype of ND88, genome-wide mining for secondary metabolite and bacteriocin biosynthetic gene clusters was performed.

The assembled ND88 genome (GenBank format) was analysed using antiSMASH v7.0 (bacterial mode) with relaxed detection stringency and default parameters. antiSMASH predicts biosynthetic gene clusters based on conserved signature domains, gene synteny, and similarity to curated reference clusters. All regions annotated as ribosomally synthesized and post-translationally modified peptide (RiPP)-like clusters, terpene clusters, type III polyketide synthase (T3PKS), and other peptide-associated clusters were extracted for further inspection. Predicted cluster boundaries and gene annotations were manually reviewed to confirm the presence of core biosynthetic genes, transport-associated proteins, and accessory modification enzymes.

To specifically identify bacteriocin-associated loci, the ND88 genome was further analysed using the BAGEL4 web server under default settings. BAGEL4 detects areas of interest (AOIs) by identifying short precursor open reading frames and conserved context genes characteristic of bacteriocin and RiPP biosynthesis. The genome sequence was uploaded in FASTA/GenBank format, and predicted AOIs were classified according to BAGEL4’s internal bacteriocin/RiPP family database.

For the plantaricin-like locus, the predicted precursor peptide, leader sequence motifs, and neighbouring genes encoding putative transport, processing, and immunity proteins were manually examined to verify its assignment as a class II plantaricin E–type bacteriocin gene cluster. The presence of conserved double-glycine leader motifs and associated ABC transporter genes was used as supporting evidence for functional annotation.

***Genome-based assessment of horizontal gene transfer potential***

To assess the potential for horizontal gene transfer (HGT) in ND88, genome annotation was performed using Prokka v1.14.5 with default parameters and species designation (“Lactiplantibacillus plantarum ND88”). Mobile genetic element–associated genes were identified from Prokka output files (GFF/TSV) by searching product descriptions using keywords including “transposase,” “insertion sequence,” “integrase,” “recombinase,” “conjugation,” and “plasmid,” followed by manual curation to exclude housekeeping recombinases. Detailed results of mobile genetic element screening are summarized in **Supplementary Table S6.**

**2. Supplementary Figures**

**Supplementary Figure S1**. **Pairwise ANI comparison among *L. plantarum, L. paraplantarum and L. pentosus* genomes including ND88.**

**Supplementary Figure S2**. Genome-based screening of acquired antimicrobial resistance genes in *Lactiplantibacillus plantarum* ND88 using the ResFinder tool (identity threshold ≥90%, minimum coverage ≥60%).

**Supplementary Figure S3.** *In silico* screening of toxigenic genes using VirulenceFinder 2.0, performed with 90% sequence identity and 60% coverage thresholds.

**3. Supplementary Tables**

**Supplementary Table S1.** **Reference genomes used for comparative genomic analyses, including ANI and pangenome analyses.**

| **Assembly (strain)** | **RefSeq accession** | **Genus Species** | **Strain (modifier)** | **ANI to ND88 (%)** | **Fragments mapped / total** |
| --- | --- | --- | --- | --- | --- |
| ASM3134858v1 | GCF_031348585.1 | *L.plantarum* | ATCC 5870 | 99.0683 | 971 / 1076 |
| ASM274965v1 | GCF_002749655.1 | *L.plantarum* | ATCC 8014 | 99.066 | 971 / 1076 |
| L_plantarum_CECT_9491_Spades_Prokka_2 | GCF_902825385.1 | *L.plantarum* | CECT 9491 | 99.0582 | 979 / 1076 |
| ASM4473190v1 | GCF_044731905.1 | *L.plantarum* | DSM 12028 | 99.0578 | 994 / 1076 |
| ASM143685v1 | GCF_001436855.1 | *L.plantarum* | DSM 13273 | 99.0429 | 987 / 1076 |
| ASM1413173v1 | GCF_014131735.1 | *L.plantarum* | DSM 20174 | 99.0281 | 984 / 1076 |
| ASM188866v1 | GCF_001888665.1 | *L.plantarum* | DSM 20246 | 99.0281 | 984 / 1076 |
| ASM2308v1 | GCF_000023085.1 | *L.plantarum* | JDM1 | 99.0189 | 985 / 1076 |
| ASM3143295v1 | GCF_031432955.1 | *L.plantarum* | KCKM 0112 | 99.0132 | 985 / 1076 |
| ASM1375388v1 | GCF_013753885.1 | *L.plantarum* | KCKM 0597 | 99.0104 | 963 / 1076 |
| ASM170431v1 | GCF_001704315.1 | *L.plantarum* | KP | 99.0104 | 975 / 1076 |
| ASM991365v1 | GCF_009913655.1 | *L.plantarum* | SRCM 100442 | 99.0052 | 991 / 1076 |
| ASM991409v1 | GCF_009914095.1 | *L.plantarum* | SRCM 101167 | 98.98 | 1009 / 1076 |
| ASM14881v1 | GCF_000148815.2 | *L.plantarum* | STIII | 98.97 | 935 / 1076 |
| ASM2156013v1 | GCF_021560135.1 | *L.plantarum* | W2 | 98.9368 | 986 / 1076 |
| ASM20385v3 | GCF_000203855.3 | *L.plantarum* | WCFS1 | 98.9248 | 959 / 1076 |
| ASM33811v2 | GCF_000338115.2 | *L.plantarum* | ZJ316 | 98.8935 | 1016 / 1076 |
| ASM14374v1 | GCF_000143745.1 | *L.plantarum* | ATCC 14917 | 98.871 | 968 / 1076 |
| ASM1835129v1 | GCF_018351295.1 | *L.plantarum* | ATCC 202195 | 98.8634 | 996 / 1076 |
| ASM1779830v1 | GCF_017798305.1 | *L.plantarum* | NCIMB8826 | 98.73 | 995 / 1076 |
| ASM364114v1 | GCF_003641145.1 | *L. paraplantarum* | DSM 10667 | 87.8435 | 826 / 1076 |
| ASM2186954v1 | GCF_021869545.1 | *L. paraplantarum* | KCCM11826P | 87.8235 | 821 / 1076 |
| ASM364118v1 | GCF_003641185.1 | *L. pentosus* | DSM 20314 | 82.8771 | 671 / 1076 |
| ASM435468v1 | GCF_004354685.1 | *L. pentosus* | ATCC 8041 | 82.8042 | 665 / 1076 |

**Supplementary Table S2**. Species-specific primers used for RT-qPCR analysis

| Bacterial strain | Primers (5’-3’) | Length (bp) |
| --- | --- | --- |
| *S. mutans* | CTACACTTTCGGGTGGCTTG | 20 |
|  | GAAGCTTTTCACCATTAGAAGCTG | 24 |
| *F. nucleatum* | AGAGTTTGATCCTGGCTCAG | 20 |
|  | GTCATCGTGCACACAGAATTGCTG | 24 |
| *P. gingivalis* | TGTAGATGACTGATGGTGAAAACC | 24 |
|  | TTTAGAGATTCGCATCCGGT | 20 |
| *P. intermedia* | TGGTTCGATAACGGCAGCAT | 20 |
|  | ACGTAGCCAACAGCAGGAAA | 20 |

**Supplementary Table S3. Genome completeness and contamination assessment of ND88**

| **Metric** | **Value** |
| --- | --- |
| BUSCO dataset | bacteria_odb10 |
| Complete BUSCOs | 123 (99.2%) |
| Single-copy | 122 (98.4%) |
| Duplicated | 1 (0.8%) |
| Fragmented | 0 (0%) |
| Missing | 1 (0.8%) |
| Total BUSCOs searched | 124 |
| CheckM marker lineage | o__Lactobacillales |
| Reference genomes | 177 |
| Marker genes | 350 |
| Marker sets | 163 |
| Completeness (%) | 98.77 |
| Contamination (%) | 0.00 |
| Strain heterogeneity (%) | 0.00 |

**Supplementary Table S4. Summary of pangenome composition and ND88-specific orthogroups identified in comparative genomic analysis.**

| **Category** | **Metric** | **Value** | **Notes** |
| --- | --- | --- | --- |
| Genome dataset | Number of genomes analyzed | 21 | ND88 + 20 reference *Lactiplantibacillus plantarum* genomes |
| Pangenome composition | Total orthologous gene clusters | 5,093 | Panaroo clustering |
|  | Core genome clusters | 2,401 | Present in all genomes (21/21) |
|  | Accessory genome clusters | 2,692 | Variably present among strains |
| ND88 genomic variation | ND88-specific orthogroups | 69 | Absent in all comparator genomes |
|  | ND88-enriched orthogroups | 15 | Increased copy number in ND88 |
| Functional annotation of ND88-specific genes | Hypothetical proteins | 64 | Majority of ND88-specific loci |
|  | IS1182 family transposase | 4 | Mobile genetic elements |
|  | Putative acetyltransferase | 1 | Predicted metabolic enzyme |
| Candidate host-interaction loci | Surface-associated orthogroups | 13 | Identified based on sequence features (LPxTG motif or signal peptide plus C-terminal TM signatures) |

**Supplementary Table S5.** PHASTER-based identification and annotation of prophage regions in ND88.

| Region | Region Length | Completeness | Score | Total Proteins | Region Position | Most Common Phage | GC % |
| --- | --- | --- | --- | --- | --- | --- | --- |
| 1 | 19.4 Kb | incomplete | 20 | 7 | 99138–101856 | PHAGE_Escher_ESCO13_NC_047770 (4) | 36.72% |
| 2 | 54.9 Kb | intact | 140 | 50 | 1755627–1810572 | PHAGE_Lactob_Sha1_NC_019489 (32) | 40.68% |
| 3 | 83.9 Kb | intact | 150 | 89 | 2089340–2173320 | PHAGE_Lactob_phi9e_NC_004305 (6) | 41.49% |
| 4 | 7.2 Kb | incomplete | 50 | 12 | 25141–32414 | PHAGE_Lactob_phiAT3_NC_005893 (2) | 37.43% |

**Supplementary Table S6.** Mobile genetic element–associated genes identified in the ND88 genome.

| **Category** | **Subtype / Gene** | **Count in ND88** |
| --- | --- | --- |
| **Transposase (IS elements)** | All transposases (total) | 15 |
|  | IS1182 family transposase | 9 |
|  | IS3 family transposase | 3 |
|  | Other IS/Tn3 transposases | 3 |
| **Tyrosine recombinase / integrase** | XerC | 5 |
|  | XerD | 1 |
|  | XerH | 1 |
|  | Prophage-like integrase | 1 |
| **Conjugation machinery** | Relaxase | 0 |
|  | Type IV secretion system components | 0 |
| **Plasmid-related genes** | Genes annotated with “plasmid” | 15 |
|  | Plasmid replicons identified | 0 |
